# Smarter eco-cities and their leading-edge artificial intelligence of things solutions for environmental sustainability: A comprehensive systematic review

**DOI:** 10.1016/j.ese.2023.100330

**Published:** 2023-10-19

**Authors:** Simon Elias Bibri, John Krogstie, Amin Kaboli, Alexandre Alahi

**Affiliations:** a School of Architecture, Civil and Environmental Engineering (ENAC), Civil Engineering Institute (IIC), Visual Intelligence for Transportation (VITA), Swiss Federal Institute of Technology Lausanne (EPFL), Lausanne, Switzerland; b Department of Computer Science, Norwegian University of Science and Technology (NTNU), Trondheim, Norway; c School of Engineering, Institute of Mechanical Engineering, Swiss Federal Institute of Technology Lausanne (EPFL), Lausanne, Switzerland

**Keywords:** Smarter eco-cities, Smart eco-cities, Smart cities, Artificial intelligence, Artificial intelligence of things, Machine learning, Environmental sustainability, Climate change

## Abstract

The recent advancements made in the realms of Artificial Intelligence (AI) and Artificial Intelligence of Things (AIoT) have unveiled transformative prospects and opportunities to enhance and optimize the environmental performance and efficiency of smart cities. These strides have, in turn, impacted smart eco-cities, catalyzing ongoing improvements and driving solutions to address complex environmental challenges. This aligns with the visionary concept of smarter eco-cities, an emerging paradigm of urbanism characterized by the seamless integration of advanced technologies and environmental strategies. However, there remains a significant gap in thoroughly understanding this new paradigm and the intricate spectrum of its multifaceted underlying dimensions. To bridge this gap, this study provides a comprehensive systematic review of the burgeoning landscape of smarter eco-cities and their leading-edge AI and AIoT solutions for environmental sustainability. To ensure thoroughness, the study employs a unified evidence synthesis framework integrating aggregative, configurative, and narrative synthesis approaches. At the core of this study lie these subsequent research inquiries: What are the foundational underpinnings of emerging smarter eco-cities, and how do they intricately interrelate, particularly urbanism paradigms, environmental solutions, and data-driven technologies? What are the key drivers and enablers propelling the materialization of smarter eco-cities? What are the primary AI and AIoT solutions that can be harnessed in the development of smarter eco-cities? In what ways do AI and AIoT technologies contribute to fostering environmental sustainability practices, and what potential benefits and opportunities do they offer for smarter eco-cities? What challenges and barriers arise in the implementation of AI and AIoT solutions for the development of smarter eco-cities? The findings significantly deepen and broaden our understanding of both the significant potential of AI and AIoT technologies to enhance sustainable urban development practices, as well as the formidable nature of the challenges they pose. Beyond theoretical enrichment, these findings offer invaluable insights and new perspectives poised to empower policymakers, practitioners, and researchers to advance the integration of eco-urbanism and AI- and AIoT-driven urbanism. Through an insightful exploration of the contemporary urban landscape and the identification of successfully applied AI and AIoT solutions, stakeholders gain the necessary groundwork for making well-informed decisions, implementing effective strategies, and designing policies that prioritize environmental well-being.

## Highlights

• AI and AIoT hold significant potential to address complex environmental challenges.
• AI and AIoT are being applied in smart cities to enhance their performance and efficiency.
• The concept of smarter eco-cities is based on the groundbreaking convergence of AI and IoT.
• Smarter eco-cities are leveraging AI and AIoT to advance their environmental sustainability goals.
• AI and AIoT pose environmental, technical, ethical, social, and regulatory challenges.

## Graphical abstract

**Figure:**
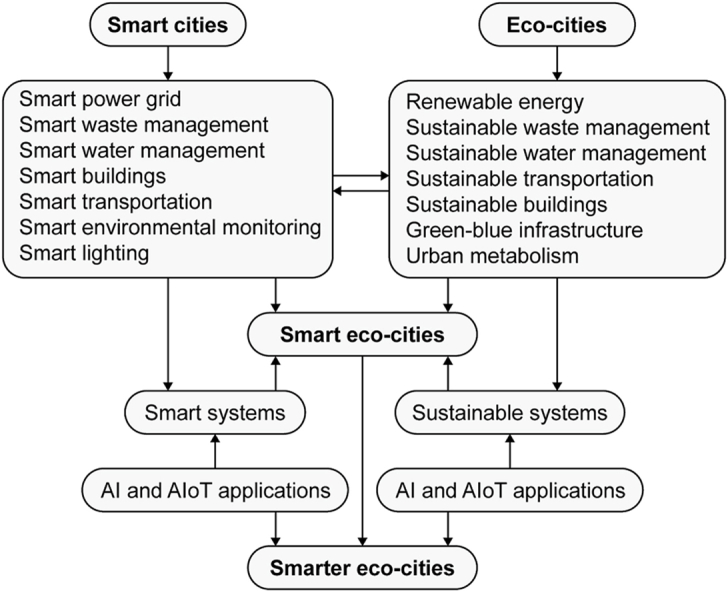

## 1 Introduction

The rapid advancement and groundbreaking convergence of Artificial Intelligence (AI) and the Internet of Things (IoT) has triggered profound transformations across various domains, including environmental sustainability, climate change, and urban development. This surge has led to the emergence of smarter eco-cities, where Artificial Intelligence of Things (AIoT) takes center stage and garners significant attention. In this context, AIoT is poised to offer innovative solutions to the mounting environmental challenges confronting smart cities and, by extension, smart eco-cities. AIoT holds the potential to unlock avenues for improving resource efficiency, reducing energy consumption, streamlining waste management, enhancing transportation management, conserving biodiversity, and mitigating environmental impacts. These advancements hold the power to reshape the urban development landscape in response to the surging wave of urbanization and the increasing complexity of ecological degradation, transforming cities into hubs of intelligence, sustainability, and environmental consciousness.

AI is rapidly reshaping both the technological and urban landscapes, serving as a pivotal driving force behind the advancement of smart cities and smart eco-cities (e.g., Ref. [[1], [2], [3], [4]]). Its potential for disruption and innovation (e.g., Ref. [5,6]) has positioned it at the forefront of these developments. This influence profoundly alters the functioning of urban systems and the intricate interactions, behaviors, and responses of their subsystems to the surrounding environment. As a result, urban processes and practices are undergoing significant transformation, marked by their increasing alignment with data-driven scientific urbanism. The impact of AI on urban systems and activities has been consistently expanding [7,8], with its computing capabilities experiencing exponential growth to accommodate the mounting influx of data from diverse sources facilitated by IoT. The potential of IoT lies in its capacity to facilitate data analysis through AI models and algorithms, a synergy poised to catalyze environmentally sustainable urban development.

The centralized infrastructure of IoT is grappling with significant challenges, primarily driven by the extensive strain imposed by the immense volume of data being generated and processed. Harnessing actionable insights from these data necessitates the integration of AI models and algorithms to effectively manage the data flow, storage, and processing inherent in the IoT infrastructure. The emergence of AIoT is underpinned by various factors that set it apart from traditional IoT. To begin with, AIoT capitalizes on the synergies between AI and IoT technologies, facilitating more intelligent and efficient data processing, analysis, and decision-making (e.g., Ref. [[9], [10], [11]]). Through the integration of AI and Machine Learning (ML)/Deep Learning (DL) capabilities into IoT devices and systems, AIoT empowers real-time data insights, predictive analytics, and adaptive responses, thereby optimizing the overall system performance and efficiency. A key distinction lies in AIoT's ability to overcome the limitations of IoT in managing the copious and diverse data generated by the vast network of interconnected devices. Additionally, AIoT addresses the challenges associated with transmitting the rapid torrent of data from distributed sensor network infrastructure [12,13]. By harnessing the power of AI, AIoT effectively processes and contextualizes intricate and multifaceted data streams, unlocking the potential for advanced applications. Most notably in certain domains, AIoT paves the way for autonomous and intelligent decision-making by IoT devices, enabling them to learn, adapt, and optimize operations in response to shifting environmental conditions and user requirements. In essence, the emergence of AIoT introduces a realm of possibilities for innovation, optimization, and automation across diverse domains, including environmental sustainability, climate change, and smart cities (e.g., Ref. [1,[13], [14], [15], [16]]).

Similar to AI, AIoT has become integral to the functioning of smart cities and, hence, smarter eco-cities. Notably, it has demonstrated innovative potential in addressing complex environmental challenges. Recent research has concentrated on the practical applications of AI and AIoT across various domains of environmental sustainability and climate change (e.g., Ref. [[17], [18], [19], [20], [21]]). Toward the end of 2020 onward, this focus has expanded to encompass smart cities in terms of their management and planning (e.g., Ref. [[22], [23], [24], [25], [26]]). Fundamentally, however, there is a strong interconnection between smart cities and eco-cities in that they have significantly influenced one another over the last decade, particularly in the domains of environmental sustainability and climate change. Eco-cities have long been associated with these two domains, serving as a well-established paradigm of sustainable urbanism (e.g., Ref. [[27], [28], [29], [30], [31], [32], [33]]). However, these two domains have frequently been addressed separately or, more recently, in connection with smart cities, particularly within the context of AI and AIoT, instead of being collectively considered within the framework of smart eco-cities. This suggests that there has been a strong tendency to prioritize one approach over another, specifically the second approach has been given a little attention. Especially, eco-cities and, by extension, smart eco-cities serve as experimental grounds for innovative solutions and environmental transitions (e.g., Ref. [27,[34], [35], [36]]). The experimentation within eco-cities extends beyond climate change to encompass energy transition, resource conservation, transport efficiency, biodiversity conservation, and experimental simulation and modeling [[37], [38], [39]]. These strategies and principles, in turn, form the fundamental driving forces behind the evolution of smart eco-cities.

Amid a world increasingly beset by uncertainties, compounded by the urgency of the climate crisis and the rapid digital transformation of smart cities and eco-cities, catalyzed by the emergence of AI and AIoT technologies, a compelling trajectory is emerging. This trajectory envisages the integration of these technologies' applied solutions into smart eco-cities to deal with the complexity of environmental degradation and climate disruption — under what can be termed as “smarter eco-cities.” This visionary concept entails the implementation of innovative, forward-looking strategies to reshape the urban landscape of the future. However, it is worth acknowledging that while AI and AIoT technologies hold immense potential yet to unlock, they pose environmental risks and amplify a spectrum of societal, ethical, legal, and regulatory challenges.

The body of literature exploring AI and AIoT solutions within the realm of environmental sustainability, climate change, and smart cities is rapidly expanding. Nonetheless, to the best of our knowledge, no review study has systematically analyzed and synthesized the existing corpus of knowledge regarding the interconnections and synergies between these three domains, coupled with their intersection with smart eco-cities with respect to AI and AIoT technologies and solutions. Furthermore, while a few recent review studies have taken a broader perspective on smart eco-cities, none have ventured into exploring their emerging technological and environmental solutions from an integrative perspective. Bridging these gaps, the present study embarks on a comprehensive systematic review of the burgeoning landscape of smarter eco-cities and their leading-edge AI and AIoT solutions for environmental sustainability. To ensure thoroughness, the study employs a unified evidence synthesis framework that seamlessly integrates configurative, aggregative, and narrative synthesis approaches. To achieve the overarching aim, the study focuses on the following specific objectives:

• Describe, illustrate, and make meaningful connections between the fundamental concepts underpinning emerging smarter eco-cities.
• Present the existing work in the field and explain how the present study differs from it and why this is a “step forward” and a new contribution to knowledge.
• Analyze, synthesize, interpret, and critically evaluate the existing knowledge in the areas of environmental sustainability, climate change, and smart cities to derive comprehensive insights into the role of AI and AIoT solutions in the advancement of emerging smarter eco-cities.
• Categorize AI and AIoT solutions based on these three areas and further evaluate their impact on advancing environmental sustainability goals.
• Capture the dynamic landscape of AI and AIoT solutions for these three areas by identifying emerging trends, innovations, and novel approaches within the framework of smarter eco-cities.
• Identify and discuss the key challenges and barriers that arise when implementing AI and AIoT solutions in the development of emerging smarter eco-cities.
• Identify existing gaps and explore relevant avenues for future research and areas requiring further investigation.

By pursuing these specific objectives, the study endeavors to provide a holistic and in-depth understanding of the integration of AI and AIoT solutions within emerging smarter eco-cities, ultimately contributing to the advancement of environmental sustainability practices in urban contexts. Toward this end, it pursues the following research questions:

RQ1: What are the foundational underpinnings of emerging smarter eco-cities, and how do they intricately interrelate, particularly urbanism paradigms, environmental solutions, and data-driven technologies?
RQ2: What are the key drivers and enablers propelling the materialization of smarter eco-cities?
RQ3: What are the primary AI and AIoT solutions that can be harnessed in the development of smarter eco-cities?
RQ4: In what ways do AI and AIoT technologies contribute to fostering environmental sustainability practices, and what potential benefits and opportunities do they offer in the realm of smarter eco-cities?
RQ5: What challenges and barriers arise when implementing AI and AIoT solutions for the development of smarter eco-cities?

By synthesizing the existing evidence and analyzing the state-of-the-art research to answer these research questions, the systematic review contributes to consolidating, enhancing, and transforming the existing knowledge on smart eco-urbanism by:

• Uncovering the dynamjc interplay between urbanism paradigms, environmental solutions, and data-driven technologies in emerging smarter eco-cities.
• Identifying the driving forces behind the materialization of smarter eco-cities, namely technological advancements, environmental concerns, and policy instruments.
• Examining the multifaceted roles of AI and AIoT in environmental sustainability, climate change, and smart cities and how these technologies can be harnessed in the development of smarter eco-cities.
• Exploring the specific ways in which AI and AIoT technologies propel sustainable development pracrices and advance environmental goals.
• Highlighting best practices where AI and AIoT solutions can significantly contribute to improving the environmental sustainability of smarter eco-cities.
• Identifying and evaluating a spectrum of challenges linked to the implementation of AI and AIoT in smarter eco-cities, aiming to illuminate potential obstacles and devise strategies to mitigate or overcome them.
• Unveiling uncharted territories and encouraging the exploration of AI and AIoT solutions to drive large-scale implementations of smarter eco-cities, propelling the discourse forward and fostering innovation in sustainable urban development.
• Providing a visionary outlook by elucidating how AI and AIoT technologies contribute to sustainable urban futures and exploring the transformative potential of these technologies in redefining urban infrastructures and systems.

In essence, the study not only presents a comprehensive overview of the current landscape of smarter eco-cities but also highlights the potential of AI and AIoT technologies in shaping the future of sustainable urban development, in addition to providing a roadmap for advancing the discourse on smarter eco-cities and facilitating interdisciplinary collaborations. Moreover, the applied unified evidence synthesis approach offers a more holistic and nuanced understanding of the research topic addressed by enhancing the thoroughness, depth, and breadth of the systematic review. The insights derived from the systematic review will not only inform researchers and practitioners in the field but also guide policymakers and practitioners in making informed decisions regarding the adoption and implementation of AI and AIoT technologies in sustainable urban management and planning. Overall, by highlighting the solutions, opportunities, benefits, and challenges in the field of smarter eco-cities, the systematic review will further facilitate the advancement of research, policy, and practice in pursuing more sustainable and technologically advanced urban environments.

This study is structured as follows: Section 2 introduces, describes, and illustrates the key conceptual strands of the study. Section 3 addresses the research review related to the study. Section 4 describes and illustrates the methodology applied in the study. Section 5 presents the results of the literature analysis and synthesis. Section 6 provides a detailed discussion, covering key challenges, open issues, and limitations. Section 7 identifies relevant gaps and presents recommendations for potential research directions and areas that require more exploration. This study concludes, in Section 8, with a summary of key findings and implications.

## 2 Conceptual background

**Fig. 1:**
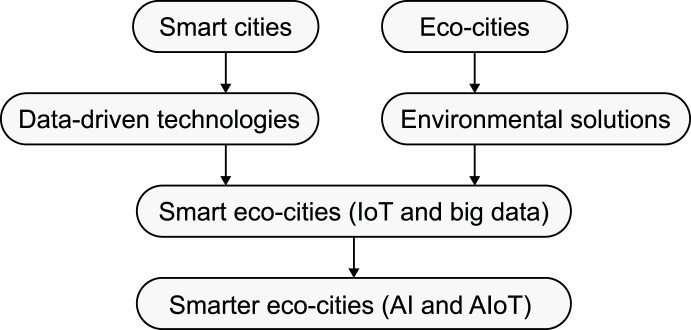
Smarter eco-cities and their underlying urbanism paradigms, environmental solutions, and data-driven technologies.

Key relevant concepts need to be clarified together with their integrative and synergistic facets. The value of linking these concepts (Fig. 1) lies in facilitating a better understanding of the foundational underpinnings of emerging smarter eco-cities in terms of urbanism paradigms, environmental solutions, and data-driven technologies.

### 2.1 Smarter eco-cities and their underlying urbanism paradigms

#### 2.1.1 Smart cities

Smart cities have gained significant attention as a potential solution to address sustainability, resource management, and urbanization challenges. Numerous attempts have been made to define the concept of smart cities. They suggest many definitions and a plethora of directions to smart city development (e.g., Ref. [[40], [41], [42]]). The concept has undergone many changes over the past two decades. In this regard, it promotes from a technology-oriented approach, i.e., infrastructures, architectures, platforms, systems, applications, and models, to a people-oriented approach, i.e., stakeholders, citizens, knowledge, services, and related data. Accordingly, it encompasses various dimensions, and there is no universally accepted definition up till now. However, the working definition for this study is justified by its alignment with the research objectives and scope. Accordingly, a smart city denotes an urban environment that leverages advanced technologies and data-driven approaches to conserve resources, minimize its environmental impact, and enhance overall ecological well-being. It prioritizes energy efficiency, sustainable transportation, waste reduction, water conservation, environmental monitoring, and green infrastructure to create a more eco-friendly and livable environment while fostering economic growth for all residents. Smart cities are increasingly emphasizing the role of technological advancements and scalable data-driven solutions to foster sustainable development practices (e.g., Ref. [[43], [44], [45], [46]]). By integrating technology with environmental stewardship, smart cities strive to create greener and healthier living environments.

However, smart cities face several challenges that need to be addressed to ensure their successful implementation as well as their integration with other emerging paradigms of urbanism. As mentioned earlier, one of the primary problems in this regard is the lack of a standardized definition for smart cities. This lack of clarity has led to confusion and inconsistency in the planning and implementation of smart city initiatives. Moreover, the existing smart city infrastructures are not designed to support the integration of advanced technologies and data-driven systems. They need to support the connectivity, data collection, and efficient management of resources, which involves scalability and interoperability. As smart cities grow and more devices and sensors are connected, their infrastructure must handle the increasing volume of data and the growing number of users. Integration and seamless communication between different systems and devices are crucial for the smooth functioning of their infrastructure. In addition, with the extensive use of data and connected devices in smart cities, ensuring the security and privacy of their infrastructure becomes paramount. These infrastructures must have robust security measures in place to protect against cyber threats and safeguard the privacy of citizens' data. Several studies (e.g., Ref. [47,48]) highlight the importance of addressing data security and privacy issues and device-level vulnerability in the context of smart cities. Furthermore, coordinating and integrating various domains to create a cohesive and integrated smart city ecosystem is a complex challenge. Smart cities should strategically use networked infrastructure and associated data-driven technologies to produce a smart economy, smart government, smart mobility, smart environment, smart living, and smart people. More so, the social, ethical, political, legal, and regulatory challenges facing smart cities have shown to be difficult to deal with. To address these challenges, a multidimensional approach focusing on technology, citizens, and institutions is necessary. This includes developing robust technology infrastructure, implementing effective governance models, and actively involving citizens in decision-making processes. Overall, while smart cities hold great potential for advancing sustainable urban development, they pose significant challenges that require a comprehensive approach to build successful and inclusive ecosystems.

#### 2.1.2 Eco-cities

The concept of eco-cities refers to “an urban environmental system in which input (of resources) and output (of waste) are minimized,” [49]. With their ubiquity today, eco-cities vary in the strategies and solutions they prioritize in response to environmental challenges in different urban contexts. They are widely diverse in conceptualization, implementation, and development. Thus, there is no definitive definition of an eco-city but rather a collection of concepts, ideas, and ambitions [32]. Broadly, eco-cities are urban areas designed and developed with a strong focus on environmental sustainability and ecological balance [50]. They aim to minimize their ecological footprint and promote a harmonious relationship between humans and nature. They prioritize energy efficiency, renewable energy sources, waste reduction, green spaces, sustainable transportation, and resource conservation. They strive to create a sustainable living environment that supports the well-being of citizens and the surrounding ecosystems while fostering social inclusivity and economic prosperity. The ultimate goal of eco-cities is to create resilient, low-carbon, and environmentally friendly urban spaces that contribute to a more sustainable future ([51,52]).

#### 2.1.3 Smart eco-cities

Eco-cities manifest themselves into different models based mainly on applying the principles of urban ecology or combining the strategies of sustainable cities and the solutions of smart cities. For the latter, the most prominent of those models are smart eco-cities, which integrate IoT and Big Data technologies with environmental technologies for achieving urban sustainability (e.g., Ref. [35,[53], [54], [55], [56]]). Smart eco-cities refer to urban environments that integrate advanced technologies, data analytics, and intelligent systems to enhance sustainability, efficiency, and quality of life while prioritizing environmental well-being. Accordingly, they leverage data-driven technologies and solutions to promote the use of renewable energy, Biomass Combined Heat Power (BCHP), sustainable transportation (walking, cycling, car sharing, biogas cars), eco-cycle waste management, green infrastructure, urban metabolism, sustainable buildings, smart grids, and sustainable urban planning strategies to minimize environmental impact, conserve resources, and foster sustainable and resilient urban environments (see Refs. [53,57] for illustrative case studies).

Unlike smart cities, smart eco-cities go beyond technology-driven approaches and emphasize environmental sustainability and ecological balance as central pillars. They focus on the integration of sustainable practices and advanced technologies, incorporate nature-based solutions into urban planning and design, and engage communities in environmental stewardship. They aim to create harmonious urban environments that not only leverage technology but also prioritize preserving and enhancing natural resources, biodiversity, and ecosystem services. In essence, they strive for a more holistic and nature-centric approach to urban development, promoting long-term sustainability and resilience and preserving natural resources for future generations to create environmentally friendly and livable urban communities.

#### 2.1.4 Smarter eco-cities

The concept of smarter eco-cities, as specific to this study, describes smart eco-cities that integrate AI and AIoT technologies and solutions with environmental technologies and strategies to maximize the performance of their sustainable systems and integrate them with smart systems given the clear synergies in their operation. This integration is intended to produce combined effects greater than the sum of the separate effects of these systems in terms of boosting the benefits of environmental sustainability. Smart city systems include smart grids, smart traffic lights, smart mobility, smart buildings, smart waste management, and smart environmental monitoring. Smarter eco-cities represent an evolution or advancement of smart eco-cities. They prioritize environmental sustainability, employing cutting-edge technologies and innovative approaches to energy, waste, water, transportation, and urban planning to create more resilient, sustainable, and technologically advanced urban environments. They also emphasize a more holistic and comprehensive approach to urban development by integrating the environmental, social, and economic dimensions of sustainability. Accordingly, they aim to balance environmental preservation, social equity, and economic prosperity. They leverage the advanced technologies and solutions offered by smarter cities, notably AI and AIoT, to optimize urban systems and address complex challenges in a more integrated and intelligent manner. They are characterized by — as derived based on the synthesized studies:

• The innovative potential of AI and AIoT technologies for sustainability;
• The enhanced sustainability outcomes enabled by the applied AI and AIoT solutions;
• The synergies between smart city systems and eco-city systems;
• The optimized performance and efficiency of smart eco-city systems; and
• The improved practices of urban management and planning.

In sum, while smart cities focus on technology-driven urban development, smart eco-cities place a stronger emphasis on achieving environmental sustainability through IoT and Big Data technologies. Smarter eco-cities go a step further by incorporating social and economic dimensions, leveraging emerging AI and IoT technologies for a more holistic approach to urban development.

### 2.2 Data-driven technologies

#### 2.2.1 IoT, computing models, and big data

The term “IoT” describes the collective network of physical objects embedded with sensing, processing, communication, and actuating technologies and capabilities that enable them to exchange data with other devices over the Internet or other networks. These objects, often called “smart devices,” can range from everyday items to complex systems like city infrastructure. IoT allows these devices to communicate and interact with each other, collect data, transfer data, and perform automated tasks, leading to enhanced efficiency, performance, and sustainability in various urban domains. More and more IoT devices are connected worldwide daily and feeding vast amounts of data into analytical systems. It is estimated that 2.5 quintillion bytes of data are being generated globally daily, which will rise to 463 exabytes by 2025 [58].

Edge, fog, and cloud computing are three interconnected paradigms that play a pivotal role in the IoT ecosystem in smart cities and smart eco-cities in the context of environmental sustainability [53]. Edge computing involves processing data at or near the data source, often within the device or a nearby gateway. It aims to reduce latency and improve real-time responsiveness by executing computations locally. It is particularly useful for applications that require quick decision-making and low-latency interactions, such as autonomous vehicles. Fog computing extends the concept of edge computing by creating a hierarchical architecture that includes multiple edge devices and gateways. Fog nodes are strategically placed in proximity to data sources to perform intermediate processing, data filtering, and preliminary analytics before transmitting relevant data to the cloud. This approach optimizes bandwidth usage and enhances system performance, making it suitable for scenarios involving distributed data sources and resource-constrained devices. Cloud computing involves using centralized remote servers to store, manage, and process data. It offers vast computational resources and storage capabilities, making it suitable for complex data analytics, machine learning, and large-scale processing. Cloud computing allows data to be accessed and analyzed from anywhere with an internet connection, making it ideal for applications that require extensive computation and storage capabilities. These computing paradigms collaborate to create a holistic IoT ecosystem that efficiently manages data processing and analysis across different levels of the network infrastructure.

Big Data refers to extremely large and complex datasets that cannot be easily managed, processed, or analyzed using traditional data processing techniques. They are huge in volume, high in velocity, diverse in variety, exhaustive in scope, fine-grained in resolution, and relational, among others. Big Data analytics involves using advanced techniques and technologies to extract valuable insights, patterns, and correlations from large and complex datasets. This process typically involves data collection, storage, processing, analysis, and visualization. In summary, IoT and Big Data are interconnected concepts revolutionizing how we collect, analyze, and utilize data. IoT enables the connectivity of smart devices, allowing them to generate vast amounts of data, while Big Data provides the means to manage, analyze, and derive meaningful insights from this data. Together, they have the potential to drive innovation, improve decision-making, and create new opportunities in diverse fields, including urban development [59].

#### 2.2.2 AI models and techniques

AI is often described as mimicking human intelligent behavior by creating computers or machines capable of its simulation. The working definition for this study describes AI as “any device/system that perceives its environment and takes actions for its goals” [60]. Broadly, an artificially intelligent machine can learn by acquiring information on the surrounding environment [61], improving performance with knowledge from experience, and performing complex tasks in a way that is similar to how humans solve problems. The capabilities of AI systems involve data analysis and learning from external data using Natural Computing (NC) and ML (e.g., Ref. [62,63]); emulating human cognitive functions using Computer Vision (CV), Fuzzy Logic (FL), Natural Language Processing (NLP) (e.g., Ref. [61]); and dealing with the complexities of human thinking and emotion (e.g., [64]) using decision support, strategic planning, sequential actions [65], self-learning, and self-improvement [66]. Concerning NC, it simulates natural phenomena and utilizes natural material as computational media in computers to optimize ML algorithms [62]. Evolutionary computing (EC) is also used for continuous optimization and in complex optimization problems involving many variables. It is extensively applied to optimize ML models [67]. For example, evolution and ecology as biological phenomena inspire optimization algorithms [68]. ML can be based on FL in terms of imitating human reasoning and cognition using 0 and 1 as extreme cases of truth with various intermediate degrees, characterizing the space between black-or-white scenarios, or using fuzzy c-means clustering to separate each data point into different clusters based on probability score attribution. CV applies ML to take information from visual data by recognizing patterns and making meaningful decisions based on that information. In addition, ML overlaps, intersects, or can be used as a tool for different AI models, such as CV, FL, and NLP.

For example, NLP enables computers to understand, analyze, manipulate, and generate human language. NLP plays a significant role in smart cities by enabling efficient and effective communication between humans and smart systems. NLP techniques analyze and understand human language in various forms, allowing for intelligent interactions and decision-making. In smart cities, NLP can be applied in multiple domains, such as smart planning, smart governance, smart mobility, and smart services. By leveraging NLP, smart cities can enhance communication channels, improve service delivery, and gain valuable insights from citizen feedback, leading to more responsive and citizen-centric urban environments. Tyagi and Bhushan [69] explore the potential of NLP in optimizing Information and Communication Technology (ICT) processes for building smart cities. The study analyzes the architecture, background, and scope of NLP and presents its recent applications in various domains. The authors highlight NLP's role in advancing smart cities and discuss the open challenges in its implementation, aiming to emphasize NLP as a key pillar in building smart cities.

Furthermore, AI involves many techniques that have gained traction over the past few years as part of AI and AIoT applications for environmental sustainability, climate change, and smart cities. These techniques include Artificial Neural Network (ANN), Support Vector Machine (SVM), Linear Regression (LR), Decision Trees (DT), Random Forests (RF), Adaptive Neuro-Fuzzy Inference System (ANFIS), Batch-Normalization (BN), Convolutional Neural Networks (CNNs), Deep Neural Networks (DNNs), and Genetic Algorithm (GA). As regards ML, among the supervised learning techniques used for regression, classification, or both are LR, Generalized Linear Models (LGM), DT, RF, SVM, ANN, and Bayesian Networks (BN). As to DL, it is a biological neural network or brain-inspired type of ML that uses DNN, CNNs, and Recurrent Neural Networks (RNNs) algorithms. Thus, it emulates the way humans gain certain types of knowledge by collecting, analyzing, and interpreting large amounts of data and making decisions in a faster and easier manner. DL techniques leverage neural networks comprising three fundamental layers: the input layer, hidden layers, and the output layer. These layers play a crucial role in acquiring data representation and establishing connections across multiple levels of abstraction.

#### 2.2.3 AIoT and its system pillars: A data science cycle perspective

To manage and analyze the dynamic and relational data generated via IoT increasingly requires powerful computational and analytical capabilities. This has led to the emergence of AIoT, a technological framework that optimizes the efficiency of IoT operations, improves human-machine interactions, advances data management and analytics models, and enhances decision-making processes. AIoT involves connecting and combining IoT devices and sensors with AI models and techniques to enable advanced analysis, enhanced real-time insights, intelligent decision-making, and autonomous behavior. It acts through control and interaction to respond to the dynamic environment, a process where ML/DL has shown value in enhancing control accuracy and facilitating multimodal interactions [13]. The synergy between AI and IoT through Big Data enables smarter and more efficient applications across various domains, driving innovation and enabling transformative solutions. IoT produces Big Data, which in turn requires “AI to interpret, understand, and make decisions that provide optimal outcomes” [70] pertaining to a wide variety of practical applications for urban systems in different context of urbanism [1,71]. In other words, IoT enables data-driven AI analytics to optimally accomplish complex tasks and extract useful knowledge in the form of applied intelligence. The resurgence of AI is driven by the abundance and potency of Big Data, thanks to enhanced computing storage capacity and real-time data processing speed.

**Fig. 2:**
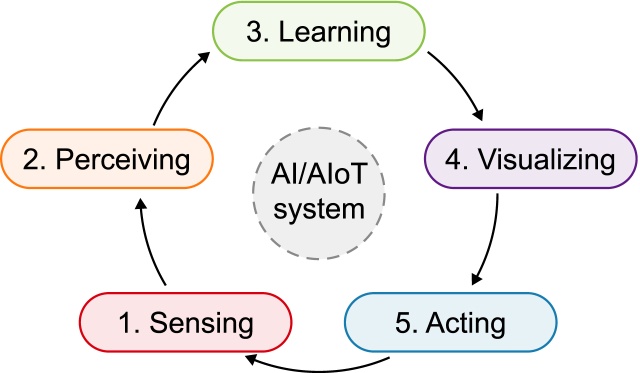
The five pillars of an AI/AIoT-driven system: 1-sensing in charge of collecting raw data, 2-perceiving in charge of extracting semantically meaningful information from raw data, 3-learning in charge of learning to predict patterns, 4-visualizing in charge of communicating key insights, and 5-acting in charge of taking action to achieve a certain goal.

AIoT enables the utilization of AI to incorporate intelligence and decision-making capabilities into IoT systems and applications. The AI/AIoT-driven system consists of five pillars: (1) sensing, (2) perceiving, (3) learning, (4) visualizing, and (5) acting. This is illustrated in Fig. 2 from a conceptually generic perspective, implying that this system can be tailored to various applications depending on their characteristics, requirements, and objectives. For example, Zhang and Tao [13] present the progress of AIoT research from four perspectives: (1) perceiving, (2) learning, (3) reasoning, and (4) behaving in connection with smart transportation, smart buildings, and smart grids. This entails empowering smart things with human-like cognitive and behavioral abilities to bring them closer to reality, which is essential to system operation.

An AI/AIoT-driven system is characterized by the ability to process raw data to extract useful insights to enable better decisions and/or take action. This involves different interrelated computational capabilities and processes. Machine perception is the capability of the system to use input data from sensors (e.g., vision, audio, proximity, position, tactile, photoelectric, infrared, light, and ultrasonic) to deduce different facets of the world, e.g., object detection/tracking, action recognition, image classification, semantic segmentation, language recognition, and pose estimation. There are different sensory information that provides patterns to the system for it to generate perceptions. Overall, machine perception aims to translate these data into meaningful information, thereby recognizing and interpreting these data by capturing the sensory information to relate to the real world. The acquisition of sensory data from the surrounding environment and their correct interpretation are key inputs to the learning process. The common state-of-the-art method for learning is based on ML, which allows the system to learn from experience without explicitly being programmed. Learning starts with collecting and preparing data (e.g., sensory, numbers, human pictures, object images, records, texts) to be used as training data, building the ML model to be trained on these data, supplying the data, and letting the system train itself to find patterns or make predictions. The outcome is an ML model that can be used with different sets of data and can use the data for predictive, descriptive, prognostic, and prescriptive functions. For example, the latter function involves suggesting what actions to take.

Further, there are three subcategories of ML: (1) supervised learning (trained with labeled data sets by humans to identify objects or things), (2) unsupervised/semi-unsupervised learning (finding patterns in unlabeled data such as behaviors or trends), and (3) reinforcement learning (trained on trial and error to take the best action based on the right decision such as autonomous driving or automating routine functions). Ullah et al. [72] illustrate different supervised, unsupervised, and reinforcement learning algorithms. These add to transduction learning, multitasking learning [73], federated learning [74], transfer learning [75], and few-shot learning [76]. Moreover, to enable the system to learn from the interpreted data and the performed computations to generate repeatable outputs and reliable decisions commonly requires using large datasets to train ML models, thereby, the relevance of DL, which can be leveraged to improve the learning of the system and enable it to adapt to varied situations to enhance its performance. DL has attracted increased attention and has proven useful for improving the intelligence of AIoT applications to handle dynamic and complex environments in the context of unsupervised and reinforcement learning methods [13].

ML is about leveraging data to improve performance on complex tasks by building decision-making models (e.g., Ref. [14,72,77,78]). Speaking of tasks, Mitchell [79] conceives of ML as “a computer program learning from experience ‘E’ with respect to some class of tasks ‘T’ and performance measure ‘P,’ if its performance at tasks in ‘T’ as measured by ‘P,’ improves with experience E.’” The workhorses of the decision-making process are ML models and algorithms, given their role in systematically extracting useful knowledge from data (patterns, correlations, predictions, forecasts, etc.) that can support decision-making. For example, by using DL algorithms, the system can conduct real-time analysis of video streams, identify objects, and detect events with absolute precision using CV models for real-time traffic monitoring or analysis of traffic conditions. The recent development of ML is associated with its ability to apply complex mathematical calculations to colossal amounts of data in a repetitive and faster manner. Accordingly, the system renders the decision-making process more data-driven, accurate, clearer, and faster thanks to ML. It makes decisions based on the perceived patterns in the data it receives. Examples of decisions in this regard may include automating a waste management process, enhancing an energy operation, optimizing a planning function, improving an environmental strategy, adjusting a policy, and evaluating risk.

As the decision-making process is based on data-driven insights, data visualization becomes important in terms of conveying complex data in such a way as to make it easier for humans to better comprehend and react to these data. Data visualization entails using specialized algorithms to generate graphical representations or visual displays of data using such elements as charts, infographics, maps, images, animations, and other metaphors. It enables decision-makers to gain and pull insights more rapidly by exploring, monitoring, and interpreting data. Examples of data visualization include city dashboards, cityScore, city metabolism, and situation rooms.

Finally, the last process of the system is acting to maximize a certain goal. To interact with the environment and humans, the system should be able to reason/make inferences and behave. Acting is associated with actuation mechanisms, which enable communication in a smarter eco-city environment and perform output functions. A wide range of actuators constitutes an integral part of the sub-systems of smarter eco-cities for operations, functions, and services. Actuation aims to execute actions to optimize different smart systems, such as power grid, building, transport, traffic, street lighting, waste management, and water distribution. The optimization occurs through adding, minimizing, adjusting, and transferring resources. In this respect, actuation is central to AIoT applications for monitoring things, controlling things, ranging things, operating things, repairing things, evaluating things, and assigning things, to name a few. Functions in smart cities “enable the actuation mechanisms to be employed directly on the IoT-enabled smart devices” [80]. For a detailed review of smart city actuators, the reader might be directed to Ref. [81]. However, developing more response systems and actuators is required to improve the engineering applications of AI and AIoT and enable their implementation in emerging smarter eco-cities concerning the performance and behavior of their physical systems.

The rationale for adopting a data science type of cycle as to the pillars of the AI/AIoT system is to emphasize the iterative and data-driven nature of the system in line with most of the synthesized studies reporting on the relationship between AIoT environmental sustainability, climate change, and smart cities. By incorporating data science principles, we seek to highlight the importance of leveraging large volumes of heterogeneous data in AIoT applications. This approach enables the extraction of meaningful insights and the development of predictive models, leading to enhanced decision-making processes and improved system performance. Highlighting the data science cycle provides a holistic AI/AIoT ecosystem perspective. It emphasizes the continuous feedback loop, where data are collected, analyzed, and fed back into the system to optimize its functionality and adaptability. This iterative process aligns with the dynamic nature of AI and AIoT applications, where data from various sources continuously flow and contribute to the intelligence and effectiveness of the overall system.

## 3 A review of related literature studies

In this section, we present a survey of the existing work conducted in the field of emerging smarter eco-cities and their applied AI and AIoT solutions. This survey aims to provide a comprehensive overview of the current state of research, highlighting the key findings, contributions, and trends in the field. By examining the existing literature, we aim to identify gaps and opportunities for further exploration in developing and implementing smarter eco-cities. This survey serves as a foundation for our comprehensive systematic review, enabling us to synthesize and analyze the findings in a structured and rigorous manner.

It was not until more recently that the literature on AI and AIoT applications for environmental sustainability and climate change started to grow and extend across many domains and disciplines. Several reviews have been performed on AI and AIoT in improving or advancing the different areas of environmental sustainability and climate change (Table 1).

**Table 1:** A set of literature review studies on AI solutions for environmental sustainability and climate change.

Areas	References
Conservation and renewable energy	[78,[82], [83], [84], [85], [86], [87], [88]]
Water resources conservation	[[89], [90], [91], [92], [93]]
Waste management	[[94], [95], [96]]
Biodiversity and ecosystem services	[[97], [98], [99]]
Sustainable transportation	[[99], [100], [101]]
Climate change adaptation and mitigation	[18,102,103,104,105]

### 3.1 AI for environmental sustainability and climate change

The review studies on energy conservation and renewable energy contribute to understanding various approaches and strategies for achieving energy efficiency and promoting renewable energy sources in different contexts. These studies highlight the importance of technological advancements, policy frameworks, and behavioral changes in achieving sustainable energy practices. In the field of water resources conservation, review studies contribute to the knowledge on water management practices, including efficient water use, water conservation strategies, and the impact of climate change on water resources. These studies emphasize the need for integrated water resource management and sustainable water use practices to address water scarcity and ensure long-term water sustainability. The research review on waste management demonstrates the significant contributions of AI in waste management, ranging from enhanced waste sorting to optimized collection routes, predictive maintenance, and Decision Support Systems (DSS). These advancements can improve resource efficiency, reduce environmental impact, and promote sustainable waste management practices. The review studies on biodiversity and ecosystem services provide insights into the importance of biodiversity conservation and the role of ecosystem services in sustaining human well-being. These studies emphasize the need for conservation measures, habitat restoration, and the integration of ecosystem services into decision-making processes for sustainable development. In the context of sustainable transportation, studies shed light on various aspects of sustainable transportation, including electric vehicles, intelligent transportation systems, and multimodal transportation options. These studies highlight the potential of sustainable transportation solutions in reducing CO_2_ emissions, improving air quality, and enhancing urban mobility. Finally, in the domain of climate change adaptation and mitigation, review studies contribute to understanding the challenges and opportunities in addressing climate change impacts. These studies explore adaptation strategies, mitigation measures, and policy frameworks to reduce GHG emissions and build resilience to climate change. Collectively, these review studies provide valuable insights into various aspects of environmental sustainability and climate change and contribute to the knowledge base in their respective fields. They highlight the importance of adopting holistic approaches, integrating multiple disciplines, and considering technological and policy dimensions to address environmental challenges and promote sustainable practices.

### 3.2 AI for smart cities

Some reviews have been conducted on the link between AI, IoT, and Big Data in smart cities from a more general perspective (e.g., Ref. [1,106,107]), broadly addressing different domains beyond environmental sustainability without providing technical details. Allam and Dhunny [106] focus mainly on the role of AI in building smart cities, addressing metabolism, governance, and culture, and identifying the strengths and weaknesses of AI. The study contributes to understanding the intersection between Big Data, AI, and smart cities. It explores the potential of utilizing these technologies in the context of smart cities, highlighting their impact on various aspects, such as resource optimization, urban planning, and transportation. It provides insights into the challenges and opportunities associated with integrating Big Data and AI in smart city initiatives. Bibri et al. [1] examine the research trends and driving factors of environmentally sustainable smart cities and their converging AI, IoT, and Big Data technologies. The authors show that environmentally sustainable smart cities have experienced rapid growth during 2016–2022, driven by both the digitalization and decarbonization agenda and the rapid advancement of data-driven technologies. The study highlights the importance of addressing the environmental costs and ethical risks associated with these technologies. The findings provide insights for scholars, practitioners, and policymakers developing data-driven technology solutions and implementing environmental policies for smart cities. Navarathna and Malagi [107] focus on the role of AI in smart city analysis. The study examines the application of AI techniques in analyzing the vast amount of data generated by smart city systems. It explores how AI can enhance decision-making processes, optimize resource allocation, and improve the overall efficiency and sustainability of smart cities. It contributes to understanding how AI can be leveraged to address smart city development challenges and complexities.

### 3.3 AIoT: theoretical foundations and practical applications

Few review studies have been carried out on AIoT as an emerging technological area. Mukhopadhyay et al. [108] highlight the importance of sensors in IoT systems and their integration with AI. The authors emphasize the need for efficient, intelligent, and connected sensors to make smart decisions and communicate collaboratively. They also mention the emergence of advanced AI technologies that enable sensors to detect performance degradation, identify patterns, and promote innovation. The focus is on sensors, smart data processing, communication protocols, and AI to enable the deployment of AI-based sensors for future IoT applications. Shi et al. [11] focus on the convergence of IoT and AI. The study compares two AI forms, knowledge-enabled AI and data-driven AI, highlighting their respective advantages and disadvantages. It surveys recent progress in the integration of AI throughout the IoT architecture, covering the sensing, network, and application layers. Zhang and Tao [13] explore the concept of AIoT and its potential to empower IoT. The study presents a comprehensive survey on AIoT, showcasing how AI techniques, particularly DL, can enhance IoT speed, intelligence, sustainability, and safety. It discusses the AIoT architecture within the context of cloud computing, fog computing, and edge computing. It also highlights promising applications of AIoT and outlines the challenges and research opportunities in this field. Both studies contribute to the understanding of the convergence of IoT and AI, with the first study focusing on the general significance and progress in the integration and the second study specifically exploring the concept of AIoT and its implications for IoT. Mastorakis et al. [12] take a broader perspective on the convergence of AI and IoT, covering various topics related to AI methods in IoT, including research trends, industry needs, and practical implementation. Their work balances theoretical concepts and real-world applications through case studies and best practices. It serves as a comprehensive resource for researchers and practitioners interested in the integration of AI and IoT. Both contributions provide insights into the integration of AI and IoT technologies and the understanding of AIoT and its applications in specific contexts and the broader IoT landscape. Together, these studies provide valuable insights into the advancements, challenges, and potential applications of AIoT, offering a foundation for further research and development in this area.

The previous review studies on AI and environmental sustainability, AI and climate change, AI and smart cities, and AIoT have laid a strong foundation for understanding the opportunities, benefits, and challenges in creating environmentally sustainable and technologically advanced urban environments. However, with the rapid advancement of AI and AIoT technologies, there is a need to explore the specific intersection of these technologies and existing smart eco-cities and their underlying multifaceted dimensions. The emerging field of AI and AIoT presents new possibilities for tackling the complexity of ecological degradation and the climate crisis in urban areas. By conducting a comprehensive systematic review of emerging smarter eco-cities and their leading-edge AI and AIoT solutions, we can bridge the gap between these solutions and the existing research on environmental sustainability, climate change, and smart cities within the defining context of smarter eco-cities. Overall, this review is the first of its kind and seeks to bring new insights into the flourishing field of smart eco-urbanism and extend the knowledge of its diverse domains by synthesizing a plethora of studies from multiple sources and disciplines.

## 4 Materials and methods

**Fig. 3:**
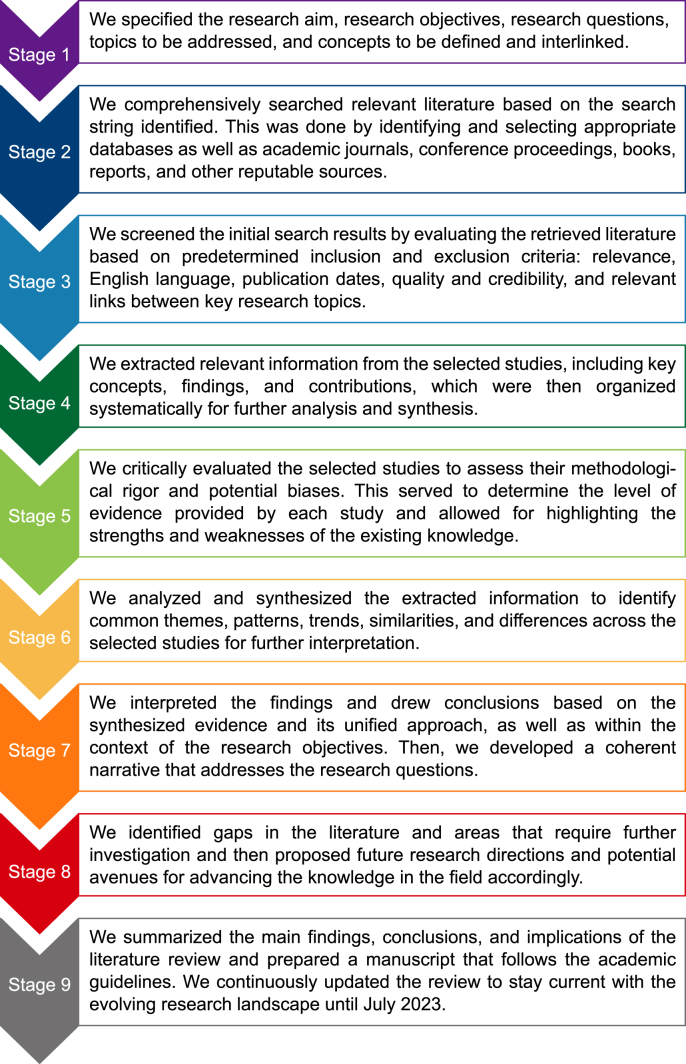
Flow diagram outlining the process of conducting the systematic review.

A systematic literature review addressed the three questions guiding the study and, hence, achieved its specific objectives. It involves retrieving, mapping, aggregating, configuring, and critically evaluating studies published to address and discuss the research topic of smarter eco-cities as an interdisciplinary field [109]. It allows for the mining of relevant information from the continuously expanding corpus of publications [110]. As illustrated in Fig. 3, the study consists of nine key stages: (1) research focus and scope definition, (2) literature search, (3) screening and selection, (4) data extraction, (5) critical evaluation, (6) synthesis and analysis, (7) interpretation and narration, (8) existing gaps and areas requiring further investigation, and (9) summary and manuscript preparation.

**Fig. 4:**
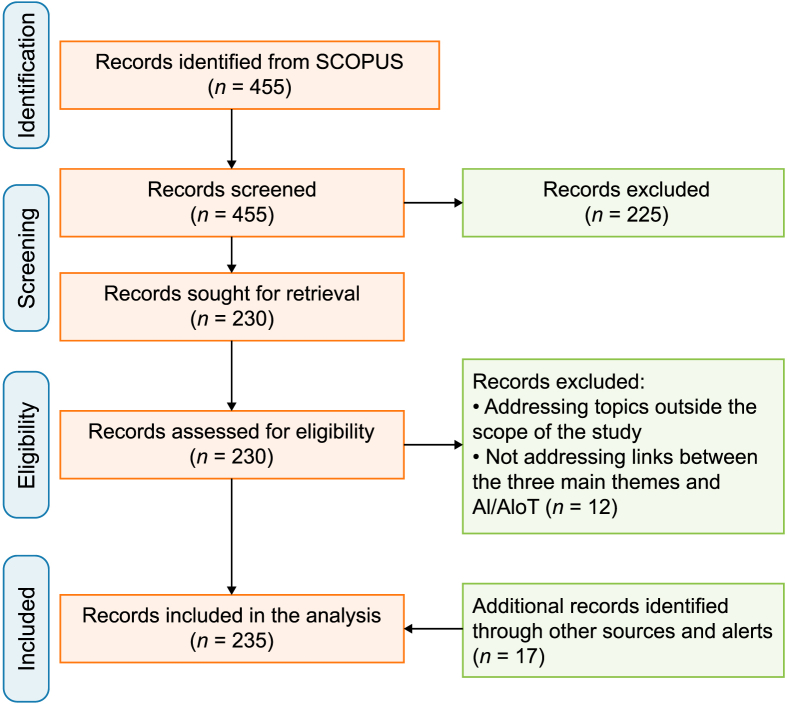
The PRISMA flowchart for literature search and selection. Adapted from [[111], [112]].

Regarding stages 2 and 3, we followed the Preferred Reporting Items for Systematic Reviews and Meta-analyses (PRISMA) approach for literature search and selection [[111], [112]]. Fig. 4 shows the four-phase flowchart literature search and selection process related to this approach. Among the available pool of academic research databases, SCOPUS was selected given its broad coverage of 455 high-quality peer-reviewed studies related to the topic on focus that meets strict standards for rigor. This online platform is one of the most reliable and trustworthy academic literature sources. To retrieve the scholarly literature, we developed a broad-based search string covering the different topics of the study and the associated links. Accordingly, the search string included: “smart eco-cities,” “smart cities AND internet of things,” “smart cities AND artificial intelligence OR machine learning OR deep learning,” “smart cities AND environmental sustainability,” “environmental sustainability AND artificial intelligence OR machine learning OR deep learning,” “climate change AND artificial intelligence OR machine learning,” “artificial intelligence of things AND environmental sustainability”, “artificial intelligence of things AND smart cities,” “artificial intelligence of things AND climate change,” “artificial intelligence AND smart eco-cities,” “blockchain AND environmental sustainability,” and “blockchain AND artificial intelligence.” These were used to search against the title, abstract, and keywords of articles to produce initial insights. We then refined and narrowed the reading scope, focusing on the documents providing definitive primary information. Accordingly, titles and abstracts from these documents were screened to select those focused on the relationships between smart cities, smart eco-cities, environmental sustainability, climate change, AI and AIoT technologies, and IoT and Big Data technologies. After excluding overlaps, 230 documents remained in the database. These were checked, and 12 papers were excluded as they did not include information on the relationships in question. Afterward, we explored citation tracking or reference chaining techniques to uncover additional relevant sources. Accordingly, the reference sections of the remaining papers were checked, and 17 other relevant papers were added to the final database, which included 235 documents in total. This was considered reliable when conducting a systematic review [113].

The reviewed articles were published in prominent journals and conferences in urban planning, sustainable urban development, computing, and emerging technologies. Among these outlets were “Sustainable Cities and Society,” “International Conference on Smart Sustainable Cities,” “IEEE Transactions on Sustainable Computing,” “Environmental Modeling and Software,” “Cleaner Production,” “Environment and Urban Systems,” “Renewable and Sustainable Energy Reviews,” “Applied Energy,” “Sustainability,” and “Technological Forecasting and Social Change.” These outlets showcased the relevance and significance of the research in advancing the understanding and implementation of AI and AIoT solutions in the context of smart eco-cities.

The literature search was conducted in late March 2023 and returned 455 documents covering 2015 to 2023. The starting year was selected because it marked the approval of the 2030 Agenda for Sustainable Development by the United Nations General Assembly as an international policy framework for the 17 SDGs. The full period, 2015–2023, captures the multifaceted nature of the topic of smarter eco-cities from the perspective of AI and AIoT technologies concerning environmental sustainability, climate change, and smart cities. This is determined by an earlier bibliometric study conducted by Bibri et al. [1], highlighting several urban trends and events highly relevant to the current study.

Data extraction and synthesis are crucial steps in conducting a systematic review. These steps involve extracting relevant information from the included studies and synthesizing the data to identify themes and patterns. Concerning stage 4, we developed a structured Excel spreadsheet that specifies what information needs to be extracted from these studies, following a deductive approach to content analysis. This information included study characteristics, methodological approaches, technological and sectoral domains, AI models and techniques applied in environmental sustainability and climate change, applied AI and AIoT solutions in smart cities, linkages between smart cities and smart eco-cities, use cases and applications, and knowledge gaps, among others. In terms of methodological approaches, for example, studies were qualitative based on descriptive analysis and literature review, mixed-methods, and quantitative approaches based on modeling and simulation regarding AI and AIoT. Moreover, we evaluated the quality and relevance of the selected documents through a critical appraisal process. This involved analyzing the methodologies used in these documents to assess the strengths and weaknesses of the research approaches. During data extraction, we carefully read and analyzed each included study to identify and record the relevant information.

**Fig. 5:**
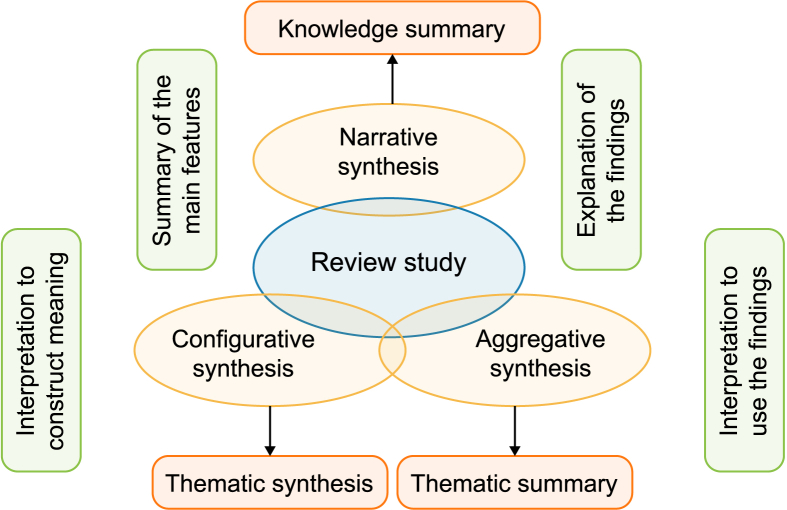
A framework for unified evidence synthesis and its characteristics.

As regards stage 5 and 6, the primary focus of this study was to identify, make, establish, and project interconnections between the different dimensions of smarter eco-cities based on the synthesized studies. The synthesized analysis involved integrating the extracted data from multiple studies to identify patterns, trends, similarities, and differences across studies to generate findings or conclusions. The synthesis approach applied seamlessly integrated configurative, aggregative, and narrative synthesis as qualitative analytical approaches. Themes were derived from the research objectives and findings from the reviewed studies. The synthesis was performed based on these themes using an integrated approach. Specific to this study, this approach attempted to strike a balance between theoretical, empirical, and practical issues. Accordingly, it included evidence from case studies, exploratory studies, observational studies, experimental studies, theory-building studies, statistical modeling studies, and review studies. The findings of the synthesized studies were merged based on a set of specified conceptual and descriptive categories. This process entailed integrating and fusing information from the multiple studies reporting on the different dimensions of emerging smarter eco-cities and related direct and indirect linkages. This categorization evolved as more precise themes were identified and revised (combined, separated, refined, or discarded). From the identified categories, themes were organized to offer new interpretations beyond the synthesized studies' findings using three different — yet complementary — approaches to synthesis (Fig. 5).

Configurative synthesis involves interpretation during the synthesis process to identify the big picture and construct the overall meaning, i.e., thematic synthesis [114]. It identifies common themes across the studies and developing a conceptual framework to explain the interconnections and variations observed in the findings. It aims to provide a deeper understanding of the research evidence by exploring the relationships and context in which the findings occur. On the other hand, aggregative synthesis involves summarizing and combining multiple research studies to produce an overall summary of the findings [115], where the interpretation is performed after the synthesis process to frame the findings, i.e., thematic summary. It aims to provide a comprehensive overview and evaluation of the research evidence. In other words, it adds and leverages evidence to make statements based on particular conceptual positions [114]. Overall, configurative synthesis goes beyond the aggregation of the research findings and focuses on understanding the underlying patterns, arrangements, or relationships among the research findings [116]. As a form of storytelling, narrative synthesis involves summarizing, explaining, and integrating the research findings from individual studies through a narrative approach. It focuses on providing a descriptive and interpretive account of the research evidence, often using textual descriptions [117]. It allows for investigating the similarities and differences between multiple studies and exploring their relationships [118]. It aims to combine diverse perspectives and findings from multiple studies to generate a coherent narrative highlighting the key concepts, themes, and implications emerging from the research. In this respect, it may also involve identifying common themes or patterns across the studies and providing an overall narrative of the findings.

## 5 Results

**Fig. 6:**
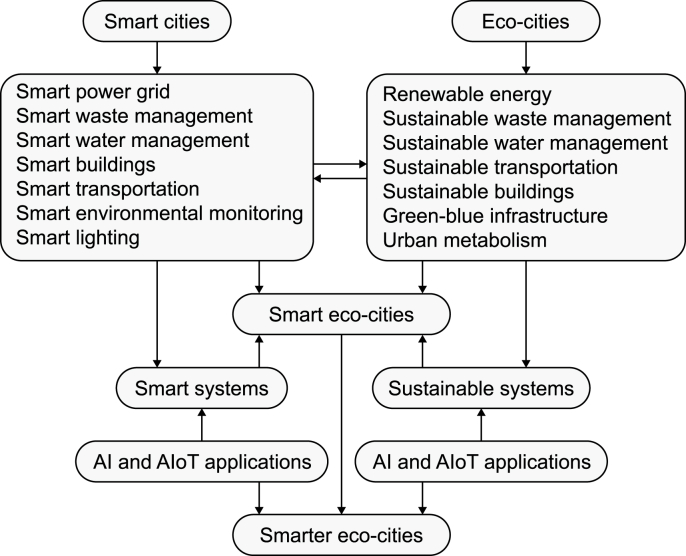
An overview of the main conceptual categories identified and their relationships.

To present the results, we consolidate all pertinent information concerning emerging smarter eco-cities as an interdisciplinary field. This consolidation encompasses a wide spectrum of theoretical, empirical, and practical evidence and pertains to the various dimensions of smarter eco-cities, highlighting their synergies in producing and enhancing environmental sustainability benefits. Fig. 6 provides a structured and navigable representation of the results.

### 5.1 The relationship between data-driven technologies, environmental sustainability, smart cities, and smart eco-cities

#### 5.1.1 On the early adoption of IoT and Big Data Technologies in smart cities in the field of environmental sustainability

To become environmentally smarter and more sustainable, both smart cities and eco-cities have undergone large-scale digital transformation enabled by the convergence of IoT, Big Data, and AI technologies. This has occurred at varying degrees and in different periods, given the specific focus of these two paradigms of urbanism in terms of strategies, solutions, and policies. Accordingly, in the early 2010s, numerous studies addressed the role of ICT in tackling the challenges of environmental sustainability in the realm of smart cities in various domains, notably:

• Power grids: to deliver energy and manage its production, consumption, and distribution to reduce costs and increase the reliability of energy supply (e.g., Ref. [119,120]).
• Environmental management: to manage natural resources and related infrastructure to improve environmental sustainability (e.g., Ref. [119,121,122]).
• Transportation management: to optimize transport efficiency and manage mobility by taking into account traffic conditions and energy usage (e.g., Ref. [121,[123], [124], [125]]).
• Waste management: to collect, recycle, reuse, recover, and dispose of different types of waste (e.g., Ref. [126,127]).

ICT enables the implementation of smart grids, which use advanced sensors, communication networks, and data analytics to optimize energy production, consumption, and distribution. This helps reduce costs, improve energy efficiency, integrate renewable energy sources, and enhance the reliability and resilience of the power grid. Moreover, ICT tools and systems facilitate the monitoring, analyzing, and managing of natural resources and related infrastructure. With sensors, remote sensing technologies, and data analytics, environmental parameters such as air quality, water quality, and waste management can be monitored and controlled in real time, enabling more effective environmental sustainability practices. Also, ICT applications contribute to optimizing transport efficiency and managing mobility in smart cities. Intelligent transportation systems, traffic management systems, and real-time data analysis help monitor traffic conditions, optimize routes, and improve energy usage. This leads to reduced congestion, better transportation planning, and reduced energy consumption and GHG emissions. Furthermore, ICT solutions are utilized to improve waste management processes. These include systems for waste collection, recycling, and disposal and technologies for waste monitoring, sorting, and tracking. By optimizing waste management operations and promoting recycling and resource recovery, ICT enables more sustainable and efficient waste management practices. ICT has played a vital role in transforming cities into smart and sustainable environments by enabling key advancements to achieve greater environmental sustainability and resilience in urban areas.

Concurrently, smart cities started to focus on embedding the next–generation of ICT into everyday objects and city structures and systems as part of the early deployment of IoT (e.g., Ref. [128,129]), paving the way for merging digital technologies with urban infrastructures and coordinating and integrating these through digital instrumentation and hyper-connectivity. One of the comprehensive theoretical and empirical studies on smart cities and IoT and Big Data conducted by Batty et al. [128] defined some goals concerning the development of a new understanding of environmental issues and the identification of critical problems relating to transport, energy, mobility, risks, and hazards. The authors additionally identified some challenges in using management, control, and optimization processes to connect smart city infrastructures to their operational functioning and planning.

During the period 2012–2015, smart cities gained traction as a model for sustainable urban development, with great potential to improve environmental sustainability based on IoT and Big Data Technologies (e.g., Ref. [[130], [131], [132], [133], [134], [135], [136]]). This traction stimulated a debate on how innovative data-driven IoT technologies could efficiently manage natural resources and mitigate environmental impacts in response to the rapid pace of urbanization and its potential effects on jeopardizing the sustainability of smart cities. Consequently, IoT and Big Data technologies gained further momentum in the pursuit of environmental sustainability across the different domains of smart cities, especially transport, mobility, energy, waste, pollution control, air quality, and planning (e.g., Ref. [[137], [138], [139], [140]]). Subsequently, they became essential to the functioning of smart cities (e.g., Ref. [40,[141], [142], [143], [144], [145]]). This was manifested in the processes and practices of smart cities becoming “highly responsive to a form of data-driven urbanism” [146]. IoT and Big Data technologies provide the ability to monitor urban operations, functions, and structures using advanced forms of decision-making in urban intelligence functions for design and planning to improve environmental sustainability.

#### 5.1.2 The influence of the IoT and Big Data Technologies of smart cities on the materialization of smart eco-cities

It is until around 2014 that smart cities started to have a significant impact on eco-cities for environmental sustainability (e.g., Ref. [57,[147], [148], [149], [150], [151]) thanks to the adoption of IoT and Big Data technologies as advanced forms of ICT. [54]) traces the evolution of smart cities and eco-cities over the past two decades regarding how their conceptual trajectories have converged under “smart eco-cities” from the mid-2010s onwards. The author highlights how this new paradigm of smart, sustainable urbanism is set to leverage the potential of IoT, Big Data, and digital infrastructures to integrate urban and green visions and policies. Smart eco-cities became widespread around 2016/2017 (e.g., Ref. [35,152]) as “a potential niche where environmental and economic reforms can be tested and introduced in areas which are both spatially proximate … and in an international context … through networks of knowledge, technology and policy transfer and learning” ([56], p. 1). Ever since, IoT and Big Data technologies and their applied solutions have become instrumental in the functioning of smart eco-cities concerning transport, mobility, energy, waste, pollution control, air quality, and planning.

To expand on the relationship between smart cities and eco-cities from an empirical perspective, Bibri and Krogstie [53] examine and compare the eco-city of Stockholm and the smart city of Barcelona, focusing on the innovative potential of IoT and Big Data technologies for advancing the goals of environmental sustainability. The authors show that smart grids, smart meters, smart buildings, smart environmental monitoring, and smart urban metabolism are the main data-driven IoT solutions adopted to enhance the performance of data-driven smart cities and smart-eco-cities under what they term “environmentally data-driven smart sustainable cities.” They also demonstrate the clear synergy between the eco-city and smart city solutions as to producing “combined effects greater than the sum of their separate effects — concerning energy efficiency and conservation improvement, environmental pollution reduction, renewable energy adoption, and real-time feedback on energy and material flows.” Pasichnyi et al. [153] propose, as part of case study research, a novel data-driven smart approach to the strategic planning of retrofitting building energy that allows a holistic city-level analysis and assesses change in total energy demand from large-scale retrofitting. Similar to Stockholm City [53], the energy transition model of Barcelona as one of the leading smart cities in Europe, aims to produce a 100 % certified renewable energy supply plan through smart energy [154]. Many other eco-cities, mainly from Europe and China, have adopted IoT and Big Data technologies in the domains of energy, transport, waste, water, and planning (e.g., Refs. [35,54,[54], [55], [56]]). A recent comprehensive state-of-the-art review conducted by Bibri [27] on smart eco-cities reveals that the newly planned and ongoing eco-city project developments are increasingly trialing innovative smart technologies to improve several aspects of environmental sustainability and climate change. This entails leveraging the advantages of eco-cities and smart cities and capitalizing on the synergies between their approaches and solutions, ultimately empowering eco-cities to enhance their environmental performance. Besides, IoT and Big Data technologies will fundamentally and irrevocably transform the landscape of eco-urbanism in terms of how eco-cities will be monitored, understood, analyzed, managed, planned, and governed.

#### 5.1.3 The influence of policy instruments and government initiatives on the materialization of smart eco-cities

The materialization of smart eco-cities is strongly influenced by, in addition to technological advancements and environmental concerns, policy instruments. Technological advancements provide the tools and capabilities for these cities to integrate and optimize various urban systems for sustainability. Environmental concerns drive the need for these cities to adopt smart and eco-friendly solutions. However, policy instruments play a crucial role in shaping and guiding the development of smart eco-cities. Policy frameworks, regulations, and incentives create an enabling environment for implementing sustainable practices and innovative technologies and ensure the effective integration of smart solutions into urban environments. The effective use of policy instruments is essential in harnessing the full potential of technological advancements and addressing environmental challenges to realize the vision of smart eco-cities. Indeed, governance and smart eco-cities are deeply intertwined in a self-reinforcing relationship. To put it differently, governance is at the core of smart eco-cities (e.g., Refs. [35,53,56,57,155]), and its key function is to make and implement policy. One of the key roles of urban policy — as a set of plans, laws, rules, regulations, and actions — lies in aligning and mobilizing the stakeholders involved in the governance of smart eco-cities. Smart eco-cities require targeted policies to drive progress in various environmental sustainability areas and effectively implement innovative data-driven solutions. Overall, policy instruments play a crucial role in the materialization of smart eco-cities by providing a framework for planning, implementing, and regulating various initiatives. Among the policy instruments that facilitate the materialization of smart eco-cities [35,55,56,[154], [155], [156]] are:

• Government regulations: The government enacts regulations that mandate specific sustainability standards and requirements for smart eco-city development, e.g., setting energy efficiency targets for buildings, enforcing waste management practices, and promoting renewable energy integration.
• Financial incentives: Governments and local authorities provide financial incentives to encourage the adoption of smart technologies and sustainable practices, including tax incentives, grants, and subsidies for smart eco-city projects that incorporate smart solutions.
• Public-private partnerships: Governments can partner with technology and energy companies, research institutions, and industry stakeholders to develop and implement sustainable solutions. These partnerships leverage expertise, resources, and funding to implement smart applications successfully.
• Open data initiatives: Governments can promote sharing data collected from various sources, such as sensors and IoT devices, to foster innovation and facilitate evidence-based decision-making. These initiatives enable researchers, businesses, and policymakers to access and analyze information to develop smart solutions and monitor the performance of smart eco-city projects.
• Standards and certification programs: Establishment of these programs ensures the interoperability, reliability, and safety of smart solutions in smart eco-cities. Standards organizations and certification bodies can define technical requirements, data privacy guidelines, and cybersecurity protocols to promote trust and facilitate the adoption of advanced technologies in smart eco-cities.

These policy instruments create a supportive environment for the materialization of smart eco-cities. They provide guidance, incentives, and regulations that drive sustainable development, promote technological innovation, and enhance the overall quality of life for residents. As revealed by Joss and Cowley [304], based on a comparative case study analysis, policy is found to exercise a strong shaping role in what sustainable development for cities is understood to be, which helps explain the considerable differences in priorities and approaches across countries.

### 5.2 The rise of AI and AIoT in environmental sustainability, climate change, and smart cities: solutions, use cases, and applications

This subsection is concerned with the solutions, applications, and use cases in the realm of AI and AIoT technologies and innovations. In this context, a solution represents a comprehensive approach encompassing software, hardware, processes, and strategies to solve a particular problem. It is implemented in real-world use cases, which depict practical scenarios illustrating how data-driven technologies work in action. On the other hand, an application refers to specific software designed to perform tasks, often utilizing both AI and AIoT models and algorithms. While these terms can overlap, they offer distinct perspectives: applications focus on software functionality, solutions provide holistic problem-solving approaches, and use cases offer practical insights into real-world implementations, collectively advancing data-driven technologies and innovations.

The use and application of AI and AIoT in environmental sustainability, climate change, and smart cities have dramatically increased from 2016 onward. This is seen as a sequel to the wide adoption of IoT and Big Data technologies and solutions in the various domains of smart cities for advancing environmental sustainability, as documented by many studies (e.g., Ref. [[137], [138], [139], [140],[143], [157]]). As presented below, the empirical, theoretical, and literature research on AI and AIoT solutions, applications, and use cases involve the different areas of environmental sustainability, climate change, and smart cities. These areas are organized into two main periods: 2016–2019 and 2020–2023, which were identified based on an earlier bibliometric study conducted by Bibri et al. [1]. This study explores the key research trends and driving factors behind the emergence of environmentally sustainable smart cities and maps their thematic evolution over time. It demonstrates the rapidly growing trend of this emerging paradigm of urbanism that markedly escalated during 2016–2022 due to the accelerated digitalization and decarbonization agendas — due to COVID-19 and the rapid advancement of data-driven technologies. Accordingly, the two periods were derived based on the accelerated digitalization of smart cities prompted by COVID-19 in early 2020 and what this entails in terms of harnessing digital technologies for addressing environmental issues (e.g., Ref. [[158], [159], [160]]), thereby the relevance of subdividing the full period into two distinct sub-periods. Also, several other studies in early 2020, as mentioned earlier, emphasized the increasing recognition of the complexity of environment degradation and climate change challenges and the growing need for more innovative, advanced, and immediate solutions based on emerging data-driven technologies to tackle them. As to the starting year of 2016, it has marked the materialization of smart eco-cities (e.g., Ref. [27,35,152]), as discussed in Section 4.

#### 5.2.1 The first period: 2016–2019

##### 5.2.1.1 Environmental sustainability

###### **Empirical AI research**

The following empirical studies have significantly contributed to the advancement of emerging smarter eco-cities by applying AI and ML models and techniques to various aspects of environmental sustainability.

• Water resources conservation: ML techniques, such as ANN, SVM, FL, ANFIS (an ANN-based on FL), LR, and Key-Nearest Neighbors (KNN), have been applied to predict stream flow and examine water quality parameters (e.g., Ref. [161,162]). Some studies have adopted ML techniques and DSS, such as ANN, DT, GA, FL, and ANFIS, to analyze water chemistry and assess water quality (e.g., Ref. [163,164]). These models and techniques have also been applied to hydro-meteorological forecasting and leak detection [[165], [166], [167]].
• Energy conservation and renewable energy: Energy-related studies have harnessed ML and DSS with techniques such as ANN, FL, SVM, DT, Evolution Strategies (ES), Evolutionary Computing (EC), BN (an algorithmic method that makes the training of DNN faster and more stable), and ANFIS for tasks including energy operation, production, distribution, maintenance, and planning (e.g., Ref. [92,161,[168], [169], [170]]).
• Sustainable transportation: ML has been applied to traffic forecasting using ANN, DT, and time series models [171,172]. Additionally, transport-related studies have employed ML and DSS with ANN, DT, NC, FL, SVM, LR, and time series models (e.g., Ref. [173,174]).
• Biodiversity conservation and ecosystem services: ML techniques, including FL, GA, ARIES, and BN (an algorithmic method used for modeling networks in ecosystems), have been used to address different aspects of biodiversity conservation and assess ecosystem services (e.g., Ref. [98,[175], [176], [177], [178], [179]]).

The insights gained from the studies conducted on water resources conservation can inform effective water resources management strategies, aiding in the conservation and sustainable use of water in existing smart eco-cities. By utilizing AI algorithms, energy conservation and renewable energy studies have contributed to energy conservation efforts and the integration of renewable energy sources within existing smart eco-cities. As to sustainable transportation, related studies have provided valuable insights for better transportation planning and management, facilitating more efficient and sustainable mobility within existing smart eco-cities. The research on biodiversity conservation and ecosystem services has contributed to a deeper understanding of the relationships between biodiversity, ecosystem services, and the sustainability of existing smart eco-cities. The findings and insights from these studies can inform evidence-based decision-making and promote sustainable practices in smart eco-city development.

###### **IoT's role within the AIoT framework**

IoT, as a fundamental component of AIoT, significantly contributes to the above areas of environmental sustainability by providing a robust technical framework for data collection, analysis, and decision-making.

• Data acquisition: IoT devices equipped with sensors and actuators collect real-time data on environmental parameters. This extensive data acquisition network forms the foundation for environmental monitoring.
• Data transmission: IoT enables seamless data transmission to centralized platforms, such as cloud computing, ensuring that data from distributed sources is readily available for processing and analysis. Connectivity protocols ensure efficient and secure data transfer.
• Big data handling: IoT generates vast amounts of data and AI algorithms, including ML and DL, process these data to identify patterns, anomalies, and trends, providing insights into environmental conditions.
• Predictive analytics: AI-driven predictive models utilize historical and real-time IoT-driven data to forecast environmental changes, which enables proactive decision-making and timely responses.
• Automation and control: IoT's integration with AI allows for autonomous control of systems and processes. For instance, smart grids can adjust energy distribution based on real-time demand and renewable energy availability, promoting energy efficiency and conservation.
• Resource optimization: AI algorithms optimize resource allocation and utilization, ensuring minimal waste and maximum efficiency in such areas as energy consumption, water usage, and transportation.
• Remote monitoring: IoT-enabled devices can be remotely controlled and monitored, reducing the need for physical interventions and minimizing human impact on sensitive ecosystems.

Overall, IoT's technical capabilities within the AIoT framework enable comprehensive data collection, analysis, and automation, fostering environmentally sustainable practices and informed decision-making across various domains.

###### **Theoretical and literature AI research**

Similarly, a range of theoretical and literature review studies have made notable contributions in the context of emerging smarter eco-cities across various domains:

• Water resources conservation: Several studies have focused on water resources conservation by applying ML and DSS, employing techniques such as ANN, SVM, FL, GA, and ANFIS. Key studies in this area include those by Mehr et al. [180], Oyebode and Stretch [90], Sahoo et al. [181], and Valizadeh et al. [182].
• Energy conservation and renewable energy: Efforts to conserve energy and promote renewable sources have been supported by ML techniques such as LR, ANN, SVM, NC, and EC. Researchers and scholars like Akhter et al. [82], Alsadi and Khatib [183], Das et al. [83], Dawoud, Lin, and Okba [184], Khan et al. [84], Youssef, El-Telbany, and Zekry [185], and Wang and Srinivasan [186] have made contributions in this field of study.
• Sustainable transportation: Related initiatives have been advanced by applying ML, CV, and DSS utilizing ANN, DT, NC, SVM, FL, and time series models. Key studies in this area include those by Jiang and Zhang [187] and Liyanage et al. [101].
• Biodiversity and ecosystem services: Biodiversity-related studies have explored modeling competition and population dynamics, often employing ML techniques like cellular automata [188]. Additionally, species conservation efforts have benefited from ML and DSS involving SVM, ANN, GA, and FL, as demonstrated by the work of Salcedo-Sanz, Cuadra, and Vermeij [97]. On the other hand, ecosystem services assessment has leveraged ML methodologies such as ARIES, as exemplified in the research by Ochoa and Urbina-Cardona [189].

The research on water resource conservation has provided insights into effective water resource management strategies. Regarding energy conservation and renewable energy, the studies in this area have contributed to optimizing energy operations, promoting energy efficiency, and fostering the adoption of renewable energy technologies. The research on sustainable transportation has provided insights into traffic management, transportation planning, and improving the overall efficiency and sustainability of transportation systems. Concerning the studies in biodiversity conservation they contribute to our understanding of biodiversity and the sustainable management of ecosystems. Overall, these theoretical and literature review studies collectively contribute to the advancement of smarter eco-cities, and their outcomes provide insights into the development of evidence-based approaches for building more sustainable and resilient smart eco-cities.

##### 5.2.1.2 Climate change: empirical AI research

In empirical AI research on climate change, notable attention has been directed toward specific areas, including scenario analysis, marine resources management, and disaster management and resilience. This focus has yielded significant contributions to advancing smarter eco-cities:

• Scenario analysis: Studies have employed various AI and ML models and techniques. Noteworthy works have performed scenario analysis based on ML, EC, and FL using ANN, BN, SVM, GA, and neuro-fuzzy (e.g., Ref. [[190], [191], [192]]); analysis across sustainability elements based on ML using ANN (e.g., Ref. [193]); and CO2 emission [[194], [195], [196]] and natural disaster (e.g. Ref. [197,198], based on ML and FL using ANN, SVM, EC, and neuro-fuzzy. Studies on ocean and cryosphere (e.g., Ref. [199]) and atmospheric forecasting (e.g., Ref. [200,201]) have applied ML using ANN. Furthermore, scenario analysis focuses on developing and analyzing different scenarios of future climate conditions based on various parameters, data inputs, and assumptions. It considers a range of potential future outcomes for climate change, including different levels of GHG emissions, temperature increases, sea-level rise, and other climate-related factors. In scenario analysis, various modeling techniques are used, including Generative Adversarial Networks (GANs), DL (CNNs and RNNs) and NLP, to create and analyze these scenarios. The goal is to provide insights into the potential consequences of different climate pathways and to help inform decision-making and policy development. Scenario analysis is valuable for understanding the range of possible future outcomes and their associated risks and impacts. It allows stakeholders to consider various “what-if” scenarios and plan accordingly for a range of potential climate-related challenges.
• Disaster management and resilience: The domain of disaster resilience has witnessed significant AI-driven contributions in prediction and forecasting and resilient infrastructure and urban planning. The studies carried out by Cheng and Hoang [305], Choubin et al. [202], and Ji et al. [203] have advanced the field by employing different AI models and techniques. Among these are ML in predictive modeling, pattern recognition, and damage assessment; NLP in sentiment analysis and information extraction; CV in image analysis and object recognition; AI-driven Geographic Information Systems (GIS) in spatial analysis and mapping and visualization; reinforcement learning in resource allocation optimization; and DSS in dynamic resource allocation.
• Marine resources management: AI research has played a pivotal role in marine resources management, encompassing water pollution monitoring, pollutant tracing in water quality, pollution reduction and prevention strategies, acidification mitigation, and habitat and species protection. These endeavors have harnessed various AI models and techniques, as demonstrated by the works of Lu et al. [204] and Wang et al. [205], to address challenges related to the sustainable use and conservation of marine ecosystems. Some of these models and techniques include ML, DL (e.g., CNNs and RNNs), GA, and ML-based Species Distribution Models (SDMs), NLP, time series forecasting, Autonomous Underwater Vehicles (AUVs) and Remotely Operated Vehicles (ROVs), and DSS.

This body of research underscores the critical role of AI in addressing climate change challenges, highlighting its potential to inform smarter eco-city initiatives and promote sustainable urban development. Specifically, the research on scenario analysis has provided insights into climate change impacts, vulnerability, and adaptation strategies; the interdependencies and interactions between different aspects of sustainability; understanding and managing CO2 emissions; and enabling the development of strategies for reducing carbon footprints. Regarding natural disaster analysis, the key insights gained pertain to predicting and forecasting natural disasters, resilient infrastructure planning, and environmental hazard detection. Disaster resilience studies have enhanced disaster resilience, enabling effective preparedness, response, and recovery measures. The studies on marine resources management have provided new perspectives on tackling different relevant challenges and enhancing the sustainable management of marine resources. Overall, these empirical studies provide valuable insights and advancements in understanding climate change challenges in the context of emerging smarter eco-cities.

##### 5.2.1.3 Smart cities

During the first period, AI research on environmental sustainability was expanding to include smart cities based on various AI models, including ML, DL, CV, NLP, and robotics (e.g., Ref. [107,[206], [207], [208], [209]]). It also involved using ML in empirical studies (e.g., Ref. [174,210]). Concerning climate change in the urban context, some studies addressed mitigation concerning urban planning, mobility, and land use (e.g., Ref. [211,212]). For AI-powered IoT architecture, research tended to focus mainly on the theoretical aspects of ML and data analysis (e.g., Ref. [[213], [214], [215]]), cognitive AI (e.g., Ref. [216,217]), knowledge-based DSS [218], and knowledge-based AI [174]. Regarding the latter, expert systems are associated with the capability of AI to solve complex problems, e.g., assessment of climate change impacts, and to aid decision-making by relying on specific knowledge derived from databases [17]. AIoT has been deployed in multiple ways to help users efficiently manage energy to reduce cost as well as energy producers optimize their equipment for better service delivery [219]. It also found important applications in vehicles and transportation, especially self-driving or autonomous vehicles. These are embedded with several sensing instruments (e.g., cameras, Light Detection and Ranging (LIDAR), and radar) and thus generate massive amounts of data [220]. LIDAR is a remote sensing technology that uses laser light to measure distances and generate precise, three-dimensional representations of objects as well as landscapes.

#### 5.2.2 The second period: 2020–2023

During the second period, 2020–2023, the empirical, theoretical, and literature research on AI increased and expanded across environmental sustainability, climate change, and smart cities. This increase and expansion pertain to energy conservation and renewable energy, water resources conservation, sustainable transport and mobility, biodiversity conservation, pollution control, climate adaptation and mitigation, and disaster management and resilience. The rapidly growing body of research in environmental sustainability, climate change, and smart cities has made further valuable contributions and advancements in the context of emerging smarter eco-cities, enhancing and extending the knowledge gained for the strategies and solutions for addressing and overcoming environmental challenges.

##### 5.2.2.1 Environmental sustainability

While the main areas of AI and AIoT research in environmental sustainability continued to attract attention, academic interest slightly decreased compared to the previous period. This is likely attributed to COVID-19 taking priority in research during the second period, inducing scholars in smart cities and environmental sustainability to investigate the link between COVID-19 and CO_2_ emissions reduction and air quality improvement. Worth pointing out is that, during 2020–2021, COVID-19-related publications received 20 % of all citations, and 98 of the 100 most-cited publications were associated with COVID-19 [1]. There was a shift during this period in academic interest from focusing on environmental sustainability challenges toward focusing on the massive deployment and implementation of digital technologies and applied solutions offered by smart cities. However, the areas of environmental sustainability that continued to attract AI research during COVID-19 include:

• Energy conservation and air quality efforts encompass pollution reduction and prevention, pollution monitoring, pollutant filtering and capture, air quality prediction, and early hazard warning, as well as the promotion of clean and renewable energy sources (e.g., Ref. [21,86,87,99,[221], [222], [223], [224]]) and environmental quality control [225].
• Sustainable transportation initiatives include the evaluation of energy consumption in household transportation through ML models [226], energy planning, transportation connectivity, urban traffic management, assessment of transport network capacity, urban traffic surveillance, and optimization of commuting corridors and jobs-housing balance using techniques, such as GA, EC, ANN, Spatial DNA, and reinforcement learning (e.g., Ref. [17,99,[227], [228], [229], [230]]).
• Clean water security and water resource conservation efforts involve various aspects such as water quality management, water supply quantity optimization, water control, water treatment, and sanitation (e.g., Ref. [93,93,231,232]). Several AI models and techniques have been applied to address challenges related to clean water security and water resource conservation, including ML, DL, ANN, CNNs, RNNs, GA, Particle Swarm Optimization (PSO), NLP, and DSS.
• Biodiversity and conservation endeavors revolve around enhancing and protecting natural capital, preserving ecosystem health, safeguarding habitats, restoring ecosystems, maintaining forest landscape visual quality, protecting species, conserving biological diversity, preventing marine pollution, and ensuring the preservation of marine resources (e.g., Ref. [99,[233], [234], [235], [236], [237],^238^]). Among the AI models and techniques being used in biodiversity and conservation efforts are ML, DL, FL, SVM, CNNs RNNs, GA, NLP, ARIES, AI-driven GIS, and AI-powered drones.

In the pursuit of creating more intelligent and sustainable urban environments, a wealth of research has emerged to address the unique challenges emerging smarter eco-cities face. These studies, spanning diverse domains, have made further contributions to shaping the trajectory of these cities. The studies on energy conservation and air quality studies significantly enhance their environmental performance and efficiency. The research in sustainable transportation research plays a crucial role in promoting eco-friendly and efficient urban transport systems. Studies on clean water security and resource conservation are pivotal for ensuring sustainable water resource management. The research on biodiversity and conservation is instrumental in supporting the development of eco-friendly and sustainable environments. These advancements can reshape the landscape of urban development models beyond smarter eco-cities in response to the growing wave of urbanization, making cities more intelligent and environmentally conscious.

##### 5.2.2.2 Climate change

Many more studies were conducted during the second period, indicating increased scholarly interest in climate change and its relation to AI and AIoT. The digitalized transformation triggered by COVID-19 significantly contributed to climate actions [158,159]. Consequently, AI and AIoT technologies gained strong traction because of the accelerated digitalization prompted by COVID-19.

###### **Mitigation and adaptation**

In the face of escalating environmental challenges, the exploration of innovative technologies has become paramount to addressing the urgent concerns of climate change. This narrative dives into the realm of climate change mitigation and adaptation, shedding light on the pivotal role that AI and AIoT play in reshaping sustainable urban development in this regard. Climate change has accelerated the need for proactive measures, particularly in urban areas where high energy consumption contributes significantly to GHG emissions. Extensive research conducted during the second period highlights the urgency of addressing climate change as cities contribute significantly to CO_2_ emissions through high energy consumption. It emphasizes the potential of AI to mitigate climate change by integrating knowledge, design strategies, and innovative technologies. It further discusses AI applications in transportation, urban energy, water use, and waste management, showcasing their impact on reshaping urban planning and design. Overall, it demonstrates that implementing AI-driven solutions can improve the sustainability of future cities and contribute to climate change mitigation efforts. Ivanova, Ivanova, and Medarov [239] acknowledge the growing influence of AI across various domains and predict its continued expansion in the coming decades. The authors emphasize the prevalence of narrow AI, specialized neural networks designed to solve specific problems, in technical fields. They focus on applying narrow AI to investigate the impact of climate change on transport infrastructure, providing guidelines for data collection and AI modeling. They highlight the controllability and capabilities of narrow AI, underscoring their potential for studying climate change's effects on transportation systems.

Furthermore, in connection with the first period, one of the areas that received more focus in the theoretical and empirical research on AI applications for climate change is disaster resilience and management, including early warning systems, resilience and planning, and simulation and prediction (e.g., Ref. [19,240,241]). However, Leal Filho [18] report on— in a systematic review and survey questionnaire — all studies conducted during 2020–2022 on the relationship between AI and climate change and its opportunities for adaptation and mitigation, covering several areas that have attracted research on AI applications. Among the themes studied by the authors in relevance to the current study while expanding on those mentioned in the first period are:

• Large-scale urbanization impacts under climate change scenarios.
• Eco-services and tradeoffs model valuation for ecosystem services quantification.
• Water utilization management using and combining Blockchain and AI.
• AI for disaster response, digital response, and disaster management.
• IoT-Based smart tree management.
• AI and ML for wildfire evacuations, wildfire prediction and prevention, wildfire susceptibility mapping, human-caused wildfire occurrence, risk-reduction strategies for floods and droughts, conservation planning under climate-changing patterns, and green-roof irrigation optimization.
• ML for flood prediction and protection.
• AI for improving resilience and preparedness against flood events impact.
• ANN for drought tolerance determination.
• Evolutionary Neural Network (ENN) for forecasting carbon emissions, energy demand, and wind generation.
• ANFIS for modeling climate change impact on wind power resources.
• DL for modeling sub-grid processes in climate models.
• ML for water security improvement and water demand forecasting in cities.
• ML for adaptation policy.
• AI for sustainable development.

Some of these themes are linked to smart cities and smart eco-cities for renewable energy, water, biodiversity, climate, planning, and policy within the framework of SDG 11: Sustainable Cities and Communities, SDG 9: Clean and Affordable Energy, and SDG 13: Climate Action. Moreover, AI can be a critical change agent because it enables climate change mitigation through carbon neutrality in energy production, distribution, transportation, buildings, construction, and others [242]. Some studies proposed benchmark datasets with additional modeling components for better climate change prediction [243]. For example, Samadi [20] notes that the convergence of AI and IoT has the potential to accurately predict floods and accelerate the convergence of AI models and techniques to advance flood analytics research. The author discusses the workflow of an AIoT prototype, namely Flood Analytics Information System (FAIS), which integrates ML, NLP, CNNs, and others.

###### **IoT's role within the AIoT framework**

In the context of climate change mitigation and adaptation, IoT as a core component of AIoT brings critical technical aspects to the forefront. As to data sensing and collection, IoT devices equipped with sensors monitor relevant environmental parameters. This continuous data stream forms the basis for understanding climate change patterns and trends. In terms of network connectivity, IoT ensures seamless data transmission and communication between devices and central data repositories. Robust communication protocols and networks, such as 5G/6G, facilitate rapid data sharing. Concerning data analysis, AI algorithms process vast datasets from IoT sensors, identifying climate change patterns and trends. ML/DL models and algorithms enable predictive analytics for anticipating changes and their potential impacts. IoT-connected weather and climate monitoring stations, coupled with AI-driven forecasting models, enable the development of early warning systems for extreme weather events and natural disasters. Moreover, IoT devices track energy consumption in real-time as part of climate change mitigation approaches. AI algorithms analyze these data to optimize energy use, identify areas for conservation, and promote the integration of renewable energy sources to reduce environmental impacts. Also, IoT-enabled infrastructure, such as smart buildings and resilient urban planning, enhances adaptation efforts. In this regard, sensors monitor structural integrity and climate-related risks, facilitating adaptive responses. Furthermore, IoT sensors in ecosystems track changes in flora and fauna behavior and health. AI algorithms aid in assessing the impact of climate change on biodiversity and guiding conservation efforts. Additionally, IoT-connected satellites and drones equipped with AI-enabled remote sensing technology provide critical data for monitoring various environmental changes. Lastly, regarding real-time decision support: IoT systems provide real-time climate data and actionable insights to decision-makers, allowing for adaptive strategies and informed policy development. In sum, IoT's technical capabilities within AIoT are instrumental in climate change mitigation and adaptation efforts. They enable data-driven decision-making, resource optimization, and resilience-building, all crucial components in addressing the challenges posed by climate change.

##### 5.2.2.3 Smart cities: transportation, energy, waste, environmental management, and the SDGs

Compared to the first period, AI, AIoT, and Blockchain technologies have proliferated and expanded, attracting more research interest, especially with their applications in smart cities. This also implies that smart cities are embracing AI and AIoT solutions developed initially for the different areas of environmental sustainability and climate change as separate fields.

###### **AI and AIoT applications**

AI and AIoT applications span many domains of smart cities in the field of environmental sustainability. In a systematic literature review on smart cities and AI, Yigitcanlar et al. [244] found that the key contributions of AI to environmental sustainability areas include:

• optimizing energy production and consumption via domotics (home automation),
• predicting the risks of climate change via ML algorithms and climate models,
• monitoring changes in the natural environment via remote sensing with autonomous drones, and
• operationalizing transport systems via mobility-as-a-service (MaaS).

AI applications related to the latter theme also include the management of transport systems of cities in terms of shared autonomous mobility-on-demand [245], autonomous cities [246], and autonomous vehicles (e.g., Ref. [[247], [248], [249]]). Concerning the latter, the deployment of AIoT at the network edge and secure trust models offer potential solutions for the real-time processing of sensor data to enable fast response to complex scenarios, such as obstacle avoidance and velocity adaptation [10]. Moreover, Zhang and Tao [13] synthesize several studies on the application of AIoT using DL in smart transportation (e.g., traffic participants, traffic infrastructures, connected logistics, and in-car driver behavior monitoring) as well as smart grids (grid fault diagnosis, building management and optimization, load monitoring and scheduling, and cyber-attack detection). To solve the load forecasting problem in energy management, Han et al. [250] propose a DL–based framework to predict future energy consumption in smart residential homes and industries, where the IoT network is connected to smart grids to maintain energy demand and supply activities effectively. The experimental results demonstrate the ability of the approach to predict energy consumption with high accuracy. El Himer et al. [251] address the role of AIoT in providing new opportunities in distributed energy resources (DER), focusing on AIoT applications in renewable energy sources, such as solar and wind. An AIoT system developed by Puri et al. [16] generates energy from different sensors, such as piezoelectric sensors, including from stress caused by human body weight, heat generated by the movement of the human body, and sunlight. The authors built and validated the data collected from the sensors with ANN and ANFIS models to predict generated power output and demonstrated that their system could produce accurate results in predicting the power generated from renewable resources.

Broadly, AIoT can be used in smart cities to analyze and track how different consumers and residents use energy to make decisions on where and what kind of renewable energy sources could be used, as well as where energy is being wasted and how it can be directed for other uses or conserved. Sleem and Elhenawy [252] discuss the contribution of AIoT to the development of smart buildings and their functionality, as well as its benefits for reducing energy consumption and costs, improving occupant comfort and productivity, and increasing safety and security. The authors also address the challenges associated with deploying AIoT and emphasize the potential of AIoT-empowered smart buildings to contribute to sustainable urban development and improve the quality of life. Furthermore, AIoT applications are converging in smart cities. Seng et al. [10] review and discuss several dimensions of AIoT applications. These are more relevant to this study — energy and smart grids, industry and smart buildings, vehicles and smart transportation, and robotics and computer vision.

Furthermore, AI and AIoT have been instrumental in developing advanced waste collection systems that optimize several parameters and maximize efficiency. Fang et al. [94] provide a comprehensive review of the application of AI in waste management, including waste-to-energy, smart bins, waste-sorting robots, waste generation models, waste monitoring and tracking, waste logistics, waste disposal, waste resource recovery, waste process efficiency, waste cost savings, and improving public health. The authors highlight the benefits of AI in waste logistics in terms of reducing transportation distance and time savings, as well as improving waste pyrolysis, carbon emission estimation, and energy conversion. They also emphasize the role of AI in increasing efficiency and reducing waste identification and sorting costs in smart cities. Nasir and Aziz Al-Talib [95] discuss the challenges in waste classification and the potential of AI and image processing techniques to address them. They acknowledge the limitations of current waste classification models driven by DL and highlight the need for improvements in accuracy and runtime to achieve precise results. They argue that accurate waste classification is crucial for multiple reasons, including enabling recycling and resource recovery, safeguarding the environment and human health, and minimizing waste management costs. The core idea distilled from the study is that waste is the byproduct of various human activities, encompassing domestic, agricultural, and industrial sectors. Different types of waste exist, including non-biodegradable, hazardous, industrial, municipal solid, and agricultural waste. Solid waste can take hundreds of years to decompose, posing environmental risks. Mounaded et al. [96] focus on applying AI techniques in municipal solid waste (MSW) management. They emphasize the use of ANN in various MSW-related problems and highlight the challenges related to data reliability and the absence of clear performance baselines for assessing AI approaches. Overall, these studies contribute to understanding how AI can revolutionize waste management by improving waste logistics, classification, and treatment processes. They highlight the potential benefits and challenges of implementing AI in the field, providing valuable insights for future research and practical applications.

In particular, the need to overcome the constraints and complexities associated with conventional approaches (e.g., RFID, GPS, GIS), especially the status and waste level in bins, has driven the development and implementation of various advanced techniques. These include PSO (e.g., Ref. [253]), ANN (e.g., Ref. [254]), and Backtracking Search Algorithm (BSA) (e.g., Ref. [255]) for waste collection optimization. GA and nearest neighborhood search algorithms have also been used for waste vehicle routing (e.g., Ref. [256]). However, these techniques still lack precision and require a long execution time. Therefore, new techniques are needed to deal with cost and emission issues and consider bin capacity, waste weight inside the bin, collection frequency, vehicle capacity and maintenance, and trip rate [257]. Further, however, AI models can be applied to predict equipment failures in waste management facilities. AI models can identify potential issues in advance by analyzing data patterns, allowing for timely maintenance and minimizing downtime. Also, AI models can enable powerful DSS for waste management. These systems integrate various data sources, including weather conditions, population density, and waste composition, to provide insights and recommendations for effective waste management strategies.

###### **Blockchain and AI and IoT applications**

Blockchain technology is gaining widespread popularity across various domains, including energy, environmental conservation, and urban development, owing to its capacity to decentralize data and processes while ensuring robust security measures. In essence, Blockchain is an open-source, peer-to-peer, distributed ledger system that encompasses multiple transactions and their associated data organized within a chain of interconnected blocks within a decentralized, peer-to-peer, and openly accessible network, using technologies such as AI, ML, and Big Data (e.g., Ref. [258]). These blocks are subject to cryptographic validation by the network itself. According to Parmentola [259], Blockchain is a rapidly evolving approach that enables the recording, sharing, updating, and synchronizing of information and transactions across multiple data ledgers or databases within a distributed and openly accessible network of diverse participants. Consequently, it fosters enhanced collaboration and interaction among various organizations and individuals participating in the network. Moreover, it is distinguished by its core attributes, including anonymity, transparency, auditability, permanence, persistence, and decentralization, which collectively translate into improved operational performance, efficiency gains, and cost reductions [258,260].

In more recent years, Blockchain has become an innovative technology and solution for smart cities in environmental sustainability. It has been used by many governments to improve environmental sustainability. Integrating blockchain into renewable energy sources can unlock energy sustainability by facilitating the development of a decentralized and democratized energy system while aiding in improved climate governance through its attributes of transparency, global decentralization, and collaborative capabilities [261]. As demonstrated in a recent bibliometric study on environmentally sustainable smart cities, Blockchain is linked to the challenges, services, and resources of smart cities and AI, IoT, and big data analytics [1]. For the latter, in a use case of a developed “blockchain-based carbon emission rights verification system,” AI and Big Data are used to learn proven data [262]. Miao et al. [263] propose a blockchain and AI-based architecture for the natural gas intelligent IoT to address the supply chain failure of existing centralized energy supply architectures because of their overwhelming numerous requests that could cause pressure, temperature, and natural gas load to exceed safety limits. Also, based on multi-sensor-driven (or IoT-based) AI tools, blockchain platforms can optimize circular economy loops ([264, 265]), allowing carbon footprint reduction and solid waste disposal control and thereby contributing to sustainability transitions [266]. Xiao et al. [267] propose using Blockchain for intelligent driving edge systems. The approach utilizes a double auction mechanism to optimize the satisfaction of users and service providers for edge computing and shows potential for better performance for resource utilization.

The value of Blockchain technology lies in storing data on green energy production activities related to environmental degradation and air pollution; enabling new means of green energy production, supply chain and logistics, real-time data collection and analysis for timely decision-making pertaining to green and low-carbon processes; and monitoring EV charging systems [259]. Moreover, Blockchain informs consumers and users about the use of less-efficient appliances. It enables them to improve their consumption behavior and thus reduce their carbon emissions [268], in addition to monitoring compliance with environmental standards by utilizing product traceability that can decrease resource inefficiencies and losses at different supply chain stages [269]. Also, Blockchain-based initiatives have been designed to provide credible trading services for polluters. They have also been used to tokenize carbon credits [269] and monitor carbon emissions. In a recent review of Blockchain applications in sustainable and smart cities, Makani et al. [270] provide a detailed account of how Blockchain technologies contribute to various urban domains, including transportation, smart grids, smart operational management, and smart homes. As regards the trading of renewable energy by local energy producers based on cryptocurrencies, Blockchain contributes to energy supply diversification, supply disruption risk reduction [269], and renewable energy promotion through microgrids and other alternative models [259]. These suggest the potential for innovative approaches that support localized and community-driven renewable energy production. By improving the alignment of energy supply and demand, these approaches can strengthen energy security and resilience, offering new avenues to enhance sustainable energy prospects. Concerning carbon emissions monitoring, the combination of Blockchain and IoT provides reliability for data and establishes measurement criteria homogeneity about registration systems and measurement tools, respectively. These solutions “minimize registration errors and eliminate fraud arising from the accounting and measurement of gas emissions” [269].

Regarding the role of AI in preventing and reducing marine pollution [271], Blockchain monitors water pollution changes and preserves marine resources [259]. It is also used in rewards schemes for residents of coastal areas using tokens of cryptocurrencies, which “can later be redeemed for credit to collect and share data on environmental conditions of water bodies” that can aid in enhancing decisions and designing regulations [269]. Similar reward schemes can raise public awareness and increase public participation in waste management and recycling by developing models to reward active users. Further, implementing Blockchain and AI as smart city technologies has several co-benefits associated with water management. Contributions in this regard relate to water provision efficiency, wastewater management, ensuring water security, groundwater monitoring, environmental awareness, promoting peer-to-peer trading of water rights, conserving water resources, and tackling the nexus between various natural resources in urban areas [259,269,271,272]. In particular, Blockchain and AI technologies could optimize water management in water-stressed urban areas by facilitating autonomous water distribution and management systems These minimize loss and control quality.

Overall, Blockchain technology, combined with AI and IoT, is crucial in advancing environmental sustainability. It provides a decentralized and transparent platform for securely recording and verifying transactions, data, and information. Integrating AI and IoT enables efficient data collection, analysis, and decision-making processes, leading to improved resource management, reduced environmental impact, and enhanced sustainability practices. The combination of blockchain, AI, and IoT allows for the development of innovative solutions such as smart grids, decentralized energy systems, waste management, and carbon footprint tracking. By fostering trust, traceability, and accountability, blockchain enhances the implementation of sustainable practices and facilitates the transition toward a greener and more sustainable future.

###### **The sustainable development goals (SDGs)**

The potential benefits of smart cities in catalyzing the transition to SDG 11 through advanced technologies and data-driven approaches are evident. Iris-Panagiota and Egleton [3] explore the role of AI within smart, sustainable cities, emphasizing its contributions to urban planning, management, and development. Zaidi et al. [273] analyze the trajectory of AI in smart sustainable cities research, pinpointing publication trends and research hotspots, including digital innovation, intelligent data systems, smart energy efficiency, and AI-IoT data analytics nexus. Yigitcanlar and Cugurullo [26] explore the sustainability of AI within the context of smart, sustainable cities, generating insights into emerging urban AI and the potential symbiosis between AI and smart, sustainable urbanism. The study reveals that AI applications have become integral in urban services, managing various aspects of urban life, such as transport systems, infrastructure, and environmental monitoring. The increasing adoption of AI is expected to continue, impacting the three dimensions of urban sustainability. Vinuesa et al. [271] reveal the potential of AI to advance 134 targets across all goals while hindering 59 targets. Collectively, these studies enrich the understanding of AI's role within smart, sustainable cities, an overarching umbrella term for smarter eco-cities — its applications in urban planning, the AI research landscape in smart cities, the integration of AI and IoT in urban contexts, the alignment of AI with SDG objectives, and the status of smarter eco-cities. Nonetheless, the trade-offs of smart cities — privacy, cybersecurity, digital divide, technology misuse, and legal frameworks — demand attention considering the use of AI and its integration (e.g., Ref. [1,3]). The imperative lies in devising measures that amplify social and economic priorities in smart city planning and development toward rendering smart eco-cities smarter and more sustainable.

## 6 Discussion: challenges, open issues, and limitations

### 6.1 Smart city AI, IoT, and Big Data Technologies as key factors impacting the dynamics of existing smart eco-cities

AI, IoT, and Big Data technologies are transforming how smart cities — and hence smart eco-cities — function by optimizing their processes, enhancing their practices, augmenting their solution capabilities, and improving their environmental sustainability performance. Since the mid-2010s, the data-driven technologies and solutions of smart cities as changing elements have gradually impacted the dynamics of eco-cities toward becoming smart in their approach to environmental sustainability by integrating their core domains with smart city domains. This will continue in the same direction as the AI, IoT, and Big Data technologies and solutions of smart cities become more advanced and integrated with sustainable technologies and strategies to provide innovative approaches that can demonstrate the ability to tackle more complex challenges. This, in turn, means making smart eco-cities smarter in their pursuit of achieving environmental sustainability thanks to the increasing use of AI and AIoT applications. The essence of AIoT revolves around the need to harness and leverage the power of smart city technologies and solutions, given the clear synergies in their operation concerning the optimization, efficiency, management, and planning processes of smarter eco-cities. This entails integrating their systems, coordinating their domains, and coupling their networks, creating many new opportunities that could be realized in environmental sustainability.

At the technical level, AI empowers the analysis of the colossal amounts of data generated via the IoT infrastructure in smart cities [14] and hence smart eco-cities, largely using ML for decision-making processes. Regarding AI-enabled sustainable smart cities, ML models can grow over time, detect invisible anomalies and alterations, exhibit various behaviors on different runs for the same input, and help provide real-time feedback for transport management, pollution control, energy management [2], and water management systems. Intelligent machines can “learn from experience, adjust to new inputs, and perform human-like tasks” ([274], p. 63) to “interpret external data correctly, to learn from such data, and to use those learnings to achieve specific goals through flexible adaptation” ([63], p. 17). Overall, AI can provide unsurpassed ways of automating or autonomizing the repetitive, complex, cognitively demanding, and time-consuming tasks associated with the operational functioning and planning of smarter eco-cities.

Concerning environmentally smart sustainable urbanism as an underlying paradigm of smart eco-cities, it is increasingly becoming a powerful societal framework for the transition toward environmental sustainability. This lies in developing joint actions for preserving the environment based on analyzing large-scale databases, understanding the complexity of climate change and modeling and simulating its potential impacts, improving the health of ecosystems, and enabling high integration of renewable energy and smart energy [271], enhancing smart renewable energy infrastructures in smart cities [275], optimizing energy consumption and production, developing more environmentally efficient transport systems, enhancing environmental monitoring (e.g., Ref. [[245], [276], [277]]), and strengthening low-carbon energy systems by supporting circular economies and smart eco-cities [271]. In particular, more than 250 studies applied AI to energy conservation and renewable energy during 2015–2019 [17]. The focus on the potential of AI and AIoT for energy can be justified by its pivotal role in the transition to smart eco-cities. This occurs through integrating large shares of renewable energy with smart energy through additional flexibility and decarbonizing other key emitting sectors, notably manufacturing, industry, transport, and buildings. Addressing the energy crisis and reducing fossil fuels will mitigate the impacts of climate change and make adaptation easier [278].

However, the dynamics of smarter eco-cities should evaluate AI and AIoT technologies as key components initiating changes in different domains. Investments in large-scale AI and AIoT as digital ecosystems are expected to positively impact smarter eco-cities that may involve feedback mechanisms, resulting in further adoption of these ecosystems and additional future investments. In other words, AI and AIoT technologies are likely to benefit from providing innovative applications in response to the need to overcome environmental sustainability challenges. Accordingly, they may exhibit positive feedback in that the more their solutions are implemented, the more likely they will be further implemented, thanks to network effects, learning, adaptation, and coordination. While the relationship between outcomes, investments, and implementations is expected to advance the transition of smart eco-cities toward environmental sustainability, stating a strong causal relationship resulting from such linkages needs to be more accurate. For this reason, coupled with other complex intertwined internal and external factors, understanding the dynamics of smarter eco-cities remains a daunting and uncertain challenge. This can be justified and elucidated in what remains of this discussion.

### 6.2 Environmental challenges and costs of AI and AIoT technologies

AI, IoT, and Big Data technologies pose significant challenges when making smart eco-cities environmentally smarter. Therefore, paying attention to both the opportunities and threats of AI and AIoT technologies is necessary. Smarter eco-cities must be environmentally friendly, thereby minimizing the negative impacts resulting from the wide use and increasing adoption of the applied solutions of AI and AIoT technologies. These enabling, integrative, and constitute technologies are embedded into a much wider socio-technical landscape involving a complex set of intertwined and heterogenous factors and actors. There is a risk of a mismatch between the environmental goals of smarter eco-cities and the opportunities offered by AI and AIoT technologies. This is due to their indirect, direct, rebound, and systemic effects, which are generated through their development, design, use, application, and disposal (see Ref. [279] for a detailed discussion). In particular, the indirect effects are expected to be exacerbated the most due to the increasing demand for AI and AIoT applications. The operation of AI and AIoT applications requires a lot of energy to power the IoT infrastructure, data processing platforms, cloud and edge computing, high-speed wireless networks, and large-scale AI systems. Concerning the latter, large data centers, which provide massive computational resources required by AI research, design, and development, are associated with significant energy consumption and, thus, carbon footprint, compromising the efforts supporting energy reduction and climate action [271]. According to current estimates, the global electricity demand for advanced ICT could increase to 20 % compared to around 1 % today [271]. AI involves establishing heavy energy dependency due to the intensive use of innovative technologies, increasing environmental impacts [[280], [281]], extending car traveling distance, and causing urban sprawl [249]. The latter two relate to exurbanization, a process whereby upper-class or affluent dwellers move from urban areas to rural areas to maintain an urban life or live in high-end housing through advanced technology or long-distance commuting.

However, the high energy requirements for AI and AIoT applications in the case of the use of non-renewable sources of energy will undermine the efforts to achieve the environmental targets of smarter eco-cities. Concerning the direct effects, building smarter eco-cities requires deploying urban operating systems, urban operations centers, and urban dashboards [53] and thus massive amounts of natural resources for developing, installing, and maintaining AI and AIoT ecosystems. In addition, IoT and AI production, distribution, service, and disposal produce vast amounts of e-waste, unsustainable materials, and toxic pollution [[63], [282]]. Almalki et al. [282] discuss, in a recent comprehensive review, the capabilities and potentials of IoT to respond to the needs of smart cities while highlighting the challenges for future research on smart city data-driven IoT applications, with a focus on their risks to environmental sustainability in terms of energy consumption, toxic pollution, e-waste, and others. All in all, the applied AI and AIoT solutions for smarter eco-cities are challenged by the effects of high energy-intensive structures, undermining the efforts deployed to avoid the overexploitation of primary resources to achieve carbon neutrality.

The green growth of AI, IoT, Big Data technologies, green computing, and eco-friendly design is critical to mitigating the risks of the mismatch between the environmental goals of smarter eco-cities and the opportunities offered by AI and AIoT technologies. This is consistent with the environment being intrinsic to SDG 11 in terms of recognizing the need to apply the most innovative technologies to make critical urban infrastructure resource-efficient, low-emission, and resilient by reaching the targets related to energy, climate, transport, waste, and water, as well as integrated policy and planning. The positive impacts of adopting sustainable approaches to the development, use, application, and disposal of AI and AIoT technologies lie in creating eco-friendly environments that are healthier and more livable in smarter eco-cities while accelerating their digital transformation. In this respect, Almalki et al. [282] analyze the various techniques and strategies for enhancing the quality of life and well-being by making cities greener, smarter, safer, and more sustainable. Bibri [[157], [279]] sheds light on the innovative role of advanced ICT as a potential remedy for mitigating its carbon footprint and thus advancing environmental sustainability goals, enabling the transition from smart cities to environmentally smarter cities. In this line of thinking, Almalki et al. [282] note that the smart things enabled by IoT in smart cities “become smarter to perform their tasks autonomously” while communicating “among themselves and humans with efficient bandwidth utilization, energy efficiency, mitigation of hazardous emissions, and reducing e-waste to make the city eco-friendly and sustainable.” Here comes the role of AI in green computing concerning smarter eco-city sensor integrated transportation systems, energy systems, building systems, waste systems, environmental monitoring systems, and so on. Green computing is key to decreasing carbon emissions and energy consumption to fulfill the environmental goals of sustainability in smart cities.

It is essential to focus on “reducing pollution hazards, traffic waste, resource usage, energy consumption, providing public safety, life quality, and sustaining the environment and cost management” to make smart cities eco-friendly [282]. This, in turn, means that AI and AIoT solutions should be carefully implemented in combination with sustainable and eco-friendly design principles, energy-efficient policy instruments, and other relevant measures. This is to ensure that the efficiency gains enabled by AI and AIoT solutions lead to reducing energy use and carbon footprint. Almalki et al. [282] provide practical insights into the data-driven IoT-based eco-friendly and sustainable cities research field. Wang and Liao [283] explore the intersection of eco-design with AI and Big Data. In doing so, they identify automation and control systems and computer science among the leading application disciplines. The authors argue for the necessity of more concerted efforts “to advance both the theoretical and empirical research on the nascent topic among researchers, funding bodies, policy-makers, and industry professionals given that the notion of eco-design of AI and Big Data applications is expected to be pertinent and relevant for designing greener strategies, products, and services for green digital transformation.” As a justification for a more consolidated green approach to AI, most attempts at using AI applications to enhance urban efficiencies have struggled, if not failed, to accomplish the transformative changes to smart cities due to “the short-sighted, technologically determined, and reductionist AI approaches being applied to complex urbanization problems” [4].

### 6.3 Technical and computational challenges of AI and AIoT technologies

Inherent to AI models and systems are several technical and analytical challenges. These include, as indicated by Nishant et al. [17] in a study conducted on AI for environmental sustainability, the overreliance of ML models on historical data, the uncertainty surrounding how humans behave in response to AI-based interventions, and the difficulty in measuring the effects of intervention strategies. Most new AI systems rely on Big Data and fail to demonstrate self-ideation or self-creation. AI must develop new concepts and models of intelligence cognition beyond ML/DL. This could offer novel solutions for environmental sustainability and climate change, feeding into new models of smarter eco-cities that may reduce decision biases due to the incompleteness and uncertainty of the data collected and aggregated in real-time. Concerning the over-reliance on Big (historical) Data in smart cities, Batty et al. (Ref. [128], p. 507) note that the prospect of real-time data collection and aggregation to deal with urban changes at any spatial or temporal scale “is a long way off and will never be reached … but what it does promise is an ability to have a real-time view of change at different spatial scales and over different time scales. This will change both the models we can build and how these technologies can inform the decision process with simulations and decision support being telescoped across space and time.” While this may be relevant to climate change in modeling and simulation, human-related variables are, to some extent, unpredictable and dynamically changing. This implies that more and varied types of data need to be collected and aggregated, new and more extensive sources of data to be explored, and new and more advanced tools for handling various velocities of data to be created. Significantly, historical datasets tend to be of limited value about climate periods and cycles, which makes it difficult to make precise predictions or decisions. A deterministic approach is difficult to adopt in climate change, as it is impossible to estimate or determine potential outcomes precisely. Non-deterministic ML models are more relevant to transport management, energy management, water management, waste management, and pollution monitoring, where they can detect anomalies and alterations and help provide real-time feedback. Furthermore, the variance-bias tradeoffs associated with ML [284] have implications for climate change solutions due to the bias and oversimplification inherent in predicting future climate change scenarios.

In addition, Kuguoglu et al. [15] investigate the reasons behind the failure of many smart city initiatives that rely on AIoT to scale up. Through a combination of literature study and expert interviews, the study identifies various factors contributing to the lack of scalability. These factors include resource and capability constraints, overlooking the importance of comprehensive change, and the influence of different factors at different stages of implementation. Regarding the technical challenges related to DL-based AIoT, they include [13]:

• Multimodal heterogeneous data processing, transmission, and storage pertaining to the massive numbers of heterogeneous sensors and the vast data streams of different formats, sizes, and timestamps they generate.
• Limited computational and storage resources in relation to using DL models for real-time data stream processing and low latency.
• Computational scheduling in AIoT architecture and related intense computation. This entails meticulous coordination across cloud centers, fog nodes, and edge devices, factoring in variables like data type, volume, network bandwidth, processing latency, performance accuracy, data security, privacy specific to the application scenario, and unbalanced data flow and user demands over time.
• Labeling unlabeled big data for DL in AIoT in terms of managing the time-consuming and financially demanding nature of this process while ensuring high-quality results.
• Data monopoly, where access to proprietary data is restricted due to vested interests, poses challenges to achieving equitable access to extensive proprietary datasets.

### 6.4 Challenges and considerations of explainable AI and interpretable ML

Explainable AI (XAI) and Interpretable ML (IML) encounter significant challenges in the context of AI and AIoT solutions for smart cities, environmental sustainability, and climate change, particularly in the evolving landscape of smarter eco-cities. XAI and IML are interconnected concepts aiming to enhance the transparency, comprehensibility, and credibility of AI models for various stakeholders involved in smarter eco-city development. While XAI focuses on explaining the decision-making process of AI systems, IML specifically concentrates on creating ML models that produce easily interpretable outcomes. XAI encompasses diverse approaches, including IML techniques, to explain AI decisions, aligning with the broader aim of enhancing explainability in AI. Both XAI and IML play pivotal roles in creating AI and AIoT systems that foster accountability, trustworthiness, and effective human-AI interaction, which is vital for making informed decisions in the context of smarter eco-cities. Nonetheless, several challenges and issues arise in this context (e.g., Ref. [[285], [286], [287], [288], [289], [290], [291],^292^]), including, but are not limited to:

• Complexity and interpretability: Applying AI and AIoT solutions to complex challenges in smarter eco-cities can lead to intricate models, hindering their interpretability. Ensuring these complex systems generate transparent decisions amid intricate environmental and climate data is crucial.
• Black-box models: Many advanced AI models (e.g., DNN) are considered black boxes, lacking transparency in decision-making. This lack of insight can hinder trust in and adoption of smart city systems, especially when critical decisions are at stake.
• Bias and fairness: Bias in AI models, derived from biased training data, can perpetuate existing inequalities in resource allocation and exacerbate environmental disparities. Overcoming these biases and ensuring fair outcomes is a daunting task. Biased decision-making becomes evident in real-time and predictive analytics, hindering the pursuit of environmental sustainability.
• The trade-off between accuracy and interpretability: Striking a balance between accurate predictions and clear explanations is essential, as more complex models might offer better predictions but sacrifice interpretability.
• Interdisciplinary nature: Addressing environmental sustainability and climate change requires expertise from diverse fields. Ensuring that AI and AIoT solutions are interpretable to domain experts, policymakers, and citizens across various disciplines is a challenge, as technical terminology can create barriers to communication.
• Data privacy and security: While explaining AI decisions and promoting transparency, care must be taken not to compromise sensitive or private information about individuals, potentially compromising their privacy.
• Dynamic and evolving environments: Smart cities and smarter eco-cities are dynamically changing environments, necessitating adaptable and robust methods for interpreting AI decisions.
• Education and adoption: Educating stakeholders, including policymakers, city planners, city managers, and citizens, about the benefits and limitations of AI and AIoT solutions, building trust and confidence, and encouraging adoption are critical factors in realizing smarter eco-cities.

The real challenge of XAI lies in granting substantial power to smarter eco-city systems without simultaneously enabling them to explain the intricate decision-making processes to different groups of domain experts. AI and ML models and algorithms assume control over decision-making by analyzing generated data, predicting outcomes, and maximizing value based on certain criteria. This reduces the rich complexity of urban life and the unpredictability of urban dynamics and systems to narrow quantitative and unitary languages, potentially disregarding the significance of cultural, ethical, social, and political values. As a result, technological advancements may pose difficulties in achieving the status of smarter eco-cities due to the mechanistic way of perceiving these complex systems. Therefore, a recent wave of research has started to focus on XAI to address some of the concerns posed by the application of AI in various domains. Mayuri, Vasile, and Indranath [290] present several applications of XAI/IML and methods to make AI/ML models explainable/interpretable. Ghonge [287] addressed several case studies and use cases of XAI as well as its impacts and challenges in smart city applications. Javid et al. [288] comprehensively delve into the landscape of XAI in smart cities, focusing on current and future developments, trends, enabling factors, use cases, challenges, and solutions. The authors outline research projects, standardization efforts, lessons learned, and technical hurdles.

XAI and IML methods are pivotal for the sustainable advancement of AI and AIoT solutions, allowing society to foster trust in the environmental and social-economic aspects of sustainability. These methods explain accuracy, fairness, transparency, accountability, and human-centeredness outcomes in AI and AIoT-powered decision-making, addressing ethical and governance concerns. These principles hold substantial relevance for data-driven decision-making in smarter eco-cities, thereby the need for creating explainable/interpretable models, techniques, and tools. Collaborative efforts among AI/AIoT experts, environmental scientists, urban planners, and policy-makers are essential to ensure the effective contribution of AI and AIoT technologies to environmental sustainability and climate change mitigation in the evolving landscape of smarter eco-cities. Also, future research endeavors will play a pivotal role in realizing transparent, effective, and ethically sound applications of XAI and IML methods within AI and AIoT solutions, advancing environmental sustainability in smarter eco-cities while ensuring equitable outcomes for all stakeholders.

### 6.5 Ethical and Societal Challenges of AI and AIoT technologies

AI technology's ethical and humanistic issues and risks are subject to long-standing intellectual and philosophical debates. The development, deployment, and adoption of AI technology raise these concerns, irrespective of the environmental benefits of its applied innovative solutions, depending on the application domain. Against the backdrop of this study, the use of AI involves making biased decisions, exacerbating privacy and cybersecurity, and limiting public trust [244,293,294]. Most of these challenges also apply by extension to AIoT, e.g., AIoT security for smart cities, AIoT and intrusion detection, and AIoT and trust recommendation [10]. Koffka [9] addresses critical concerns, including security and privacy, interoperability, and ethics, underscoring the importance of a responsible AIoT ecosystem. Furthermore, using AI entails devaluating human abilities, deepening information asymmetries, undermining equal power relations, and causing system failures. Regarding the latter, increasing public awareness of this type of risk is crucial before launching large-scale AI deployments in a society increasingly dependent on AI technology [271]. This indeed is arcane in that its actual functionalities and mechanisms are understood by only a group of people, despite being already part of the everyday life of many of us [6]. Therefore, given that AI as a disruptive technology will greatly transform smart eco-cities, it needs to earn public trust regarding how people perceive it. Also, AI technology needs to gain the trust in the minds of government agencies and public organizations investing in it [295]. The challenges posed by AI generally involve gaps in ethical standards, including safety, fairness, transparency [293,296], socio-economic equality, cultural diversity, and social inclusion. For example, safety is a key topic in ethical and legal debates over autonomous systems [297,298]. Most ethical issues raised by AI and AIoT applications relate to the difficulties in explaining AI models or interpreting ML algorithms, which resemble black boxes, with some being the hardest for humans to comprehend. There is a need for developing new methods for Explainable AI (XAI) or Interpretable ML (IML) that allow humans (designers, engineers, researchers, city planners, city managers, regulators, and policymakers) to understand and trust the decisions or predictions that AI models and systems make in terms of their potential biases and expected impacts. Ghonge [287] addressed several case studies and use cases of XAI as well as its impacts and open challenges in smart city applications.

The underlying ethical gaps of AI technology call for designing and implementing appropriate regulatory frameworks to address the counterproductive outcomes emanating from the penetrative patterns of AI (e.g., Ref. [63,244,299,300]) in urban life domains in emerging smarter eco-cities. Especially, early in the decision-making process of its deployment — when the opportunity for effective inputs and informed choices is greatest. This pertains to developing “responsible and ethical AI” before it is too late [297,[301], [302]]. There is a warrant for this as the integration of AI with IoT and Big Data is speeding up the pace of advancements and innovations in both AI and AIoT systems, particularly the exponential rise of their computational power, paving the way for them to gain more and more power of the automation and autonomization of smart cities, with profound implications for smarter eco-cities. While it is possible to automate certain urban processes and practices concerning environmental sustainability and climate change, it is necessary to carefully plan and implement them to avoid generating fully automated or autonomous smarter eco-cities based on mechanical decisions. In this regard, it is essential to address and overcome the regulatory challenges pertaining to the use of AI and AIoT applications to advance environmental sustainability. Vinuesa et al. [271] emphasize the need for regulatory insight and oversight to support the development of AI-based technologies for sustainable development.

The realization of the common good of AI and AIoT technologies remains highly improbable when AI systems operate solely according to the algorithms designed and implemented by powerful corporations driven by ambitions for power, profit, and extensive reach and influence. These tech giants pursue various trajectories and explore uncharted possibilities, raising concerns about the potential consequences of the large-scale implementation of AI and AIoT systems. There is an urgent demand for well-regulated and responsible AI and AIoT systems that prioritize safeguarding public and civic values within the context of smart eco-cities. Such systems must be designed to ensure that the broader benefit to society takes precedence over corporate interests and unchecked advancements. Indeed, civic values and public values play vital roles in the functioning of both civil society and government. These values are the moral compass that guides individuals, communities, and public institutions in their pursuit of a just, inclusive, and prosperous society. In a civil society, civic values, such as social justice, freedom, tolerance, compassion, and tolerance, are the cornerstones of a harmonious and fair community. They inspire individuals to engage in civic activities, advocate for their rights, and work collectively to address societal issues. Civic engagement is fostered by these values, encouraging citizens to participate in public discourse and actively contribute to the betterment of society. At the same time, public values are fundamental to the proper functioning of government. These principles, including accountability, transparency, integrity, inclusivity, public participation, and environmental stewardship, ensure that public institutions operate in the best interests of the people they serve. However, for example, in relation to environmental stewardship, the crucial aspects of environmental protection, justice, and preservation are often sidelined when unregulated economic interests drive urban development.

While technological advancements, such as AI and AIoT systems, have the potential to enhance the efficiency and effectiveness of both civil society and government, it is essential to recognize that certain core functions should not be outsourced to these systems. The decision-making processes guided by civic and public values require the nuanced judgment and ethical considerations that only humans can provide. AI and AioT systems can be valuable tools, but they should support and complement the efforts of individuals and institutions rather than replace or overshadow the importance of these foundational values. The interplay between civic and public values and emerging technologies should be carefully managed to ensure that they continue to serve as the moral and ethical foundations of our society and government. However, Kassens-Noor and Hintze [303] argue that the adoption rate of AI technology, coupled with policy regulations and unforeseen events, has the potential to transform bustling metropolises into deserted ghost cities. The complete advancement of AI and AIoT may signify a decline in moral and societal values, raising concerns about the potential demise of the human race. Nevertheless, despite the enticing conceptual and discursive benefits (which relate to both ideas, theories, and perceptions, as well as the ways in which they are discussed and communicated) of transitioning cities into eco-cities, formidable obstacles have hindered large-scale implementations since the early 1990s, not to mention the development of smart(er) eco-cities. One of the most significant challenges confronting urban transformations lies in the significant costs, risks, and uncertainties associated with the incorporation of AI and AIoT into the realm of eco-urbanism.

### 6.6 Methodological limitations

It is important to acknowledge the methodological limitations of this comprehensive systematic review to allow the readers to assess the reliability and validity of the findings and understand the potential implications for future research and practice. These limitations, which arose from the various aspects of the review process, include:

• Search strategy: Despite efforts to conduct a thorough literature search, some relevant studies may have been missed. Limitations in database selection, search keywords, language restriction, and inclusion/exclusion criteria could influence the breadth and depth of the included studies for synthesis. Moreover, as the study relied mainly on peer-reviewed documents, there is a risk of excluding a large part of grey literature and stakeholder input and not gaining extensive insights on emerging smarter eco-cities. This area of research is still evolving, and most of its existing work drew mainly from the fields of environmental sustainability, climate change, and smart cities with respect to AI and AIoT technologies and solutions. Even these fields are associated with a paucity of knowledge and a sparsity of empirical evidence due to the burgeoning nature of these technologies and solutions.
• Publication bias: The study relied on published literature, and there is a risk of publication bias, where studies with positive results are more likely to be published, while studies with negative findings may be overlooked. This can affect the comprehensiveness and representativeness of the systematic review. It is common for researchers and journals to preferentially publish studies based on the direction or significance of their findings. This can lead to an overrepresentation of studies with positive results, while studies with negative results may be less likely to get published and thus be included in the systematic review. In addition, there is a language bias in that studies published in English are more accessible and commonly included in systematic reviews, leading to the potential exclusion of valuable evidence. Especially, English language was one of the inclusion criteria applied in the study. Funding sources are another bias regarding the studies funded by industry having a higher likelihood of being published — if they produce favorable results.
• Data extraction and synthesis pertain to the complexities and challenges of extracting data from selected studies and synthesizing their findings. One of the primary issues posed in the study was the heterogeneity across studies. This included variations in study designs (e.g., methodologies) that complicated the synthesis of findings and reporting formats (differences in the presentation of results) that affected the extraction and synthesis process. These differences can make it difficult to directly compare and combine data from different studies, thus obtaining a comprehensive overview of the evidence and drawing robust conclusions from the systematic review.

## 7 Suggestions for future research

Smarter eco-city scholars, practitioners, and policymakers have a new opportunity to foster sustainable development practices based on a new paradigm of solution-thinking grounded in a deeper understanding of the interplay between techno-scientific and socio-political solutions. Developing this multi-faceted change process is one of the most critical challenges of sustainable urban development to achieve the status of smarter eco-cities. This emerging area of research is empirically under-researched, theoretically under-developed, and critically under-thought to allow for large-scale implementations. This means that a plethora of problems and questions need to be addressed and answered to guide the development of smarter eco-cities and, hence, large-scale AI and AIoT deployments for the common good. Some gaps in our knowledge of emerging smarter eco-cities follow from our results and discussion. These gaps span a broad set of topics that are significant to investigate or critically engage with and that can be approached from various perspectives in the form of suggestions for future research.

Considering the transformative potential of AI and AIoT technologies in reshaping smarter eco-cities, a comprehensive investigation is warranted to unravel the dimensions, opportunities, benefits, and challenges inherent in this emerging urban paradigm. Given the nascent stage of research at the intersection of environmental sustainability, climate change, and smart cities within the context of AI and AIoT solutions, the following avenues are crucial:

• Identify key drivers: Delve into the multifaceted drivers that underpin the evolution of smart eco-cities, encompassing social, economic, institutional, and political factors beyond technological and environmental aspects.
• Evaluate effectiveness: Prioritize assessing the real-world effectiveness and scalability of AI and AIoT applications in smarter eco-cities, determining their actual impact on achieving SDGs.
• Explore long-term benefits: Probe the long-term benefits and opportunities offered by AI and AIoT technologies in fostering sustainability practices in smarter eco-cities. This includes exploring their potential for resource optimization, energy efficiency, waste reduction, and enhancing the quality of life for citizens.
• Overcome barriers and risks: Confront challenges and mitigate risks associated with AI and AIoT implementation, such as privacy concerns, data security, and governance frameworks.
• Promote responsible AI practices: Investigate guidelines and best practices for the responsible design, deployment, and governance of AI and AIoT solutions. Ensuring fairness, transparency, and accountability in decision-making processes is essential.
• Integrate disciplines: Foster interdisciplinary research merging environmental sustainability, climate change, and smart cities with AI and AIoT. This approach unravels intricate relationships and facilitates a comprehensive understanding of the complex interactions and interdependencies between these domains, and enables the development of integrated and holistic solutions
• Formulate policies and frameworks: Develop robust policy and governance frameworks that facilitate the ethical and transparent use of AI and AIoT technologies and support their adoption and implementation in sustainable urban development. This includes examining regulatory mechanisms, standards, and guidelines to ensure transparency, accountability, and ethical use of these technologies.
• Advance XAI and IML methods: Develop solutions for interpretability in intricate models, user-centric model training, tailored solutions for smarter eco-cities, resilience and adaptability enhancement, and ethical implications. This entails developing XAI techniques for elucidating complex AI models in AIoT systems, exploring IML integration to engage users in refining models, utilizing XAI and IML to tackle distinct environmental challenges, fortifying AIoT systems' resilience against dynamic urban scenarios, and scrutinizing the ethical and societal implications of deploying XAI and IML in AIoT solutions to ensure equity and transparency.
• Promote community engagement: Explore ways to involve local communities, stakeholders, and citizens in the design, implementation, and monitoring of AI and AIoT solutions for smarter eco-cities. Their active participation can lead to more inclusive and effective outcomes.
• Quantify environmental impact: Develop methodologies to quantify the environmental impact of AI and AIoT solutions in smarter eco-cities. This involves assessing factors like energy consumption, carbon footprint, and resource utilization to understand the overall sustainability gains.
• Conduct lifecycle analysis: Assess the sustainability of AI and AIoT technologies across their entire lifecycle, from production to disposal. This holistic approach can reveal potential environmental hotspots and guide improvements.
• Promote citizen participation: Explore ways to empower citizens with AI-augmented information and tools that enable them to participate in environmental conservation and sustainable behaviors actively.
• Enhance behavioral insights: Investigate how AI can leverage behavioral insights to encourage environmentally friendly behaviors among citizens, such as energy conservation and waste reduction.
• Perform case studies: Conduct in-depth empirical inquiries and practical applications of AI and AIoT in actual smart eco-city projects. These real-world insights illuminate challenges, best practices, and valuable lessons for effective integration.

By addressing these knowledge gaps and pursuing these research avenues, the field of smarter eco-cities can advance its understanding, implementation, and impact. This will contribute to developing more sustainable and technologically advanced urban environments that benefit society and the environment.

## 8 Conclusion

As disruptive technologies, AI and AIoT lay the foundational technological infrastructure essential for constructing the digital ecosystem of emerging smarter eco-cities to amplify and sustain their contributions to environmental sustainability goals. This pursuit involves enhancing the efficiency and effectiveness of their operations, functions, strategies, and policies in alignment with the environmental targets of SDG 11. Within this context, it is important to acknowledge the immense potential of AI and AIoT technologies to develop robust intelligent systems generating profound insights for decision-making processes. However, these technologies cannot serve as a universal remedy or panacea for the wicked problems characterizing smarter eco-cities as complex systems. In this study, we aimed to provide a comprehensive systematic review of emerging smarter eco-cities and their leading-edge AI and AIoT solutions for environmental sustainability, employing a unified approach to evidence synthesis. The study's key findings concerning the five research questions are outlined as follows:

Interlinked foundational underpinnings of smarter eco-cities: The study showed that the fundamental concepts underpinning smarter eco-cities are intricately interconnected and build one on another on various scales. The key underlying urbanism paradigms, namely smart cities and eco-cities, serve as the foundation for integrating data-driven technologies and environmental solutions. Data-driven technologies enable real-time monitoring, analysis, and decision-making, while environmental solutions focus on optimizing resource efficiency and minimizing ecological footprint. Data-driven insights enhance the effectiveness of environmental strategies, ultimately contributing to creating more resilient, livable, and environmentally friendly urban environments.

The materialization of smarter eco-cities: The study identified several intertwined factors contributing to the materialization of smarter eco-cities as an emerging paradigm of urbanism, including the growing need for sustainable development, advancements in technology, environmental considerations, policy instruments, and government initiatives, and the recognition of the potential of data-driven technologies in addressing complex environmental challenges.

The primary AI and AIoT solutions harnessed in the development of emerging smarter eco-cities: The study identified many applied solutions of AI and AIoT technologies, demonstrating their role in urban planning, management, and development. These solutions encompass energy conservation and renewable energy, sustainable transportation management, traffic control, water resources conservation, waste management for efficient resource utilization, biodiversity and ecosystem services, environmental monitoring and control, climate change adaptation and mitigation, and disaster resilience and management.

The benefits and opportunities of AI and AIoT technologies in fostering sustainability practices in emerging smarter eco-cities: The study identified the opportunities and benefits offered by AI and AIoT technologies in the context of environmental sustainability. Combined, these opportunities and benefits included optimized resource management, increased energy efficiency, enhanced waste management, improved transportation, and mobility management, reduced environmental impacts, increased resilience to environmental challenges, enhanced decision-making in urban management and planning, and the potential for creating more sustainable and technologically advanced urban environments. Identifying opportunities — favorable circumstances and possibilities — helps understand the potential areas where AI and AIoT solutions can bring about positive changes and contribute to the overall development of smarter eco-cities. Benefits — positive outcomes and advantages — highlight the tangible and intangible gains that can be achieved by adopting AI and AIoT solutions.

Challenges and barriers arising in the implementation of AI and AIoT solutions for the development of emerging smarter eco-cities: The study identified and evaluated the key challenges pertaining to environmental costs, privacy concerns related to data collection and usage, cybersecurity risks related to interconnected systems, public trust, and social acceptance, limited technical expertise and knowledge, the lack of robust regulatory frameworks to ensure ethical and responsible AI and AIoT deployment and the requirement for addressing the social issues to ensure equitable and transparent use of AI and AIoT technologies.

Overall, the study highlighted the significance of AI and AIoT technologies in advancing the transition toward environmental sustainability in smarter eco-cities. While these technologies provide new and largely expanded opportunities to understand better and prevent environmental problems, they pose significant challenges that must be addressed and overcome to successfully implement smarter eco-cities. Therefore, it is important to emphasize the need for interdisciplinary research, policy support, and collaboration among different stakeholders to overcome these challenges and maximize the benefits of these technologies.

The synthesized evidence presented in this study has significant implications for researchers, practitioners, and policymakers involved in designing, managing, and planning smarter eco-cities. It offers valuable insights into the various dimensions of emerging smarter eco-cities and identifies best practices that can inform decision-making processes. The systematic review serves as a knowledge repository, guiding stakeholders in understanding the current state of research, identifying gaps, and shaping future strategies for sustainable urban development. Firstly, by identifying the core conceptual underpinnings of emerging smarter eco-cities and the intricate interconnections between them (RQ1), coupled with the intertwined factors propelling the materialization of smarter eco-cities (RQ2), the study encourages interdisciplinary collaboration among researchers and practitioners from various fields and foster new research avenues and practice pathways, leading to a more thorough understanding and focused improvement of emerging smarter eco-cities. Secondly, by identifying the key applied solutions of AI and AIoT technologies (RQ3), the study can inform researchers, practitioners, and policymakers about the technological advancements and innovative approaches employed in fostering sustainable urban development practices. Furthermore, exploring the potential opportunities and benefits offered by AI and AIoT technologies in this regard (RQ4) can provide valuable insights for decision-makers and urban planners seeking to leverage these technologies for achieving the SDGs, especially SDG 11. Lastly, identifying challenges and barriers in implementing AI and AIoT solutions in emerging smarter eco-cities (RQ5) can inform policymakers and stakeholders about the potential obstacles and open issues that need to be addressed when integrating these technologies into urban development strategies. Overall, the research, practice, and policymaking implications of this study encompass a wide range of areas, including urban planning, technology implementation, sustainability practices, and policy development, facilitating informed decision-making, and promoting the advancement of smarter eco-cities.

Ultimately, the findings of the systematic review contribute to the broader goal of creating smarter eco-cities that prioritize environmental sustainability, resource efficiency, and human well-being. The invaluable insights gained accordingly will empower stakeholders to make strategic choices, implement innovative solutions, and drive positive change in urban planning and management. By leveraging the potential of AI and AIoT, policymakers, urban planners, researchers, and practitioners can work together toward creating smarter, more resilient, more livable, and environmentally conscious cities that meet the needs of present and future generations. To sum up, AI and AIoT technologies will offer unprecedented capabilities to rise to many of the grand environmental challenges, but how these technologies will be used and what other possible directions this use might take is up to all of us, especially the research community, and for the time to tell.

## CRediT authorship contribution statement

**Simon Elias Bibri**: Conceptualization, Methodology, Formal Analysis, Investigation, Data Curation, Visualization, Software, Writing - Original Draft, Writing - Review & Editing. **John Krogstie**: Conceptualization, Writing - Review & Editing. **Alexandre Alahi** and **Amin Kaboli**: Writing - Review & Editing. All authors read and approved the published version of the manuscript.

## Declaration of competing interest

The authors declare that they have no known competing financial interests or personal relationships that could have influenced the work reported in this article.

## Abbreviations

AI: Artificial Intelligence
AIoT: Artificial Intelligence of Things
ANFIS: Adaptive Neuro-Fuzzy Inference System
ANN: Artificial Neural Network
ARIES: Artificial Intelligence for Ecosystems
AUVs: Autonomous Underwater Vehicles
BC: Bayesian Classifier
BN: Batch-Normalization
CNNs: Convolutional Neural Networks
CO_2_: Carbon Dioxide
COVID-19: Corona Virus Disease 2019
CV: Computer Vision
DL: Deep Learning
DNN: Deep Neural Networks
DSS: Decision Support Systems
DT: Decision Trees
EC: Evolutionary Computing
ENN: Evolutionary Neural Network
ES: Evolutionary Strategies
FAIS: Flood Analytics Information System
FL: Fuzzy Logic
GA: Genetic Algorithm
GANs: Generative Adversarial Networks
GIS: Geographic Information Systems
ICT: Information and Communication Technology
IML: Interpretable Machine Learning
IoT: Internet of Things
KNN: K-Nearest Neighbour
LIDAR: Light Detection and Ranging
Maas: Mobility-as-a-service
ML: Machine Learning
NC: Natural Computing
NLP: Natural Language Processing
RNNs: Recurrent Neural Networks
ROVs: Remotely Operated Vehicles
SDGs: Sustainable Development Goals
SDMs: Species Distribution Models
SVM: Support Vector Machine
XAI: Explainable Artificial Intelligence
